# Revitalizing Micro‐Sized Si‐Based Anodes Through Advanced Structural Design and Interface Stabilization: A Review

**DOI:** 10.1002/smll.202506603

**Published:** 2025-07-31

**Authors:** Luwen Li, Qitao Shi, Zhipeng Wang, Jiaqi Wang, Cheng Zhang, Junjin Zhang, Xiangqi Liu, Alicja Bachmatiuk, Yanbin Shen, Chen Lu, Ruizhi Yang, Mark H. Rümmeli

**Affiliations:** ^1^ College of Energy Soochow Institute for Energy and Materials Innovation Key Laboratory of Advanced Carbon Materials and Wearable Energy Technologies of Jiangsu Province Key Laboratory of Core Technology of High Specific Energy Battery and Key Materials for Petroleum and Chemical Industry Soochow University Suzhou 215006 P. R. China; ^2^ i‐Lab CAS Center for Excellence in Nanoscience Suzhou Institute of Nano‐Tech and Nano‐Bionics (SINANO) Chinese Academy of Sciences (CAS) Suzhou 215123 P. R. China; ^3^ Faculty of Chemistry Wrocław University of Science and Technology Wybrzeże Wyspiańskiego 27 Wrocław 50—370 Poland; ^4^ Electron Beam Emergent Additive Manufacturing (EBEAM Centre) Centre for Nanotechnlogy (CNT) Centre for Energy and Environmental Technologies (CEET) VSB—Technical University of Ostrava 17 Listopadu 15 Ostrava 708 33 Czech Republic; ^5^ School of Electronic and Information Engineering Changshu Institute of Technology Changshu 215500 P. R. China; ^6^ Jiangsu Key Laboratory of Advanced Negative Carbon Technologies Soochow University Suzhou Jiangsu 215123 P. R. China; ^7^ Key Laboratory of Core Technology of High Specific Energy Battery and Key Materials for Petroleum and Chemical Industry Soochow University Suzhou 215006 P. R. China

**Keywords:** advanced characterization techniques, binder modifications, electrolyte designs, micro‐sized Si‐based anodes, structural designs

## Abstract

Silicon (Si) is recognized as a promising anode material for next‐generation lithium‐ion batteries owing to its exceptionally high lithium storage capacity. Recently, micro‐sized Si (micro‐Si) based anodes have re‐emerged as alternatives to nano‐sized Si (nano‐Si) owing to their higher tap density and reduced interfacial side reactions. Considerable efforts are devoted to addressing the rapid capacity decay caused by severe volume expansion, sluggish kinetics, and continuous accumulation of the solid electrolyte interphase. In this review, the primary failure mechanisms of micro‐Si anodes is first analyzed and subsequently summarize recent advances in enhancing their structural and interfacial stability. The design of Si‐containing materials (primarily Si/C composites and SiO_x_ structures) that meet the current industrial requirements is discussed. Additionally, binder optimization and electrolyte exploration are analyzed. Finally, the potential application of advanced spectroscopic, electronic, and mechanical characterization techniques is explored, coupled with machine learning, in developing Si‐based anodes. This review aims to comprehensively understand the rational design and in‐depth analysis of next‐generation micro‐Si based lithium‐ion batteries.

## Graphical abstract

This review outlines strategies to enhance micro‐Si anode structural integrity via tailored designs, micro‐Si, Si/C, SiO_x_, optimized binders/electrolytes, and advanced characterization. Machine learning integration further guides solutions, advancing the science of high‐energy lithium‐ion batteries with durable cycling stability.

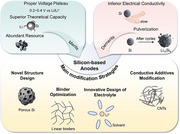

## 1 Introduction

**Figure 1:**
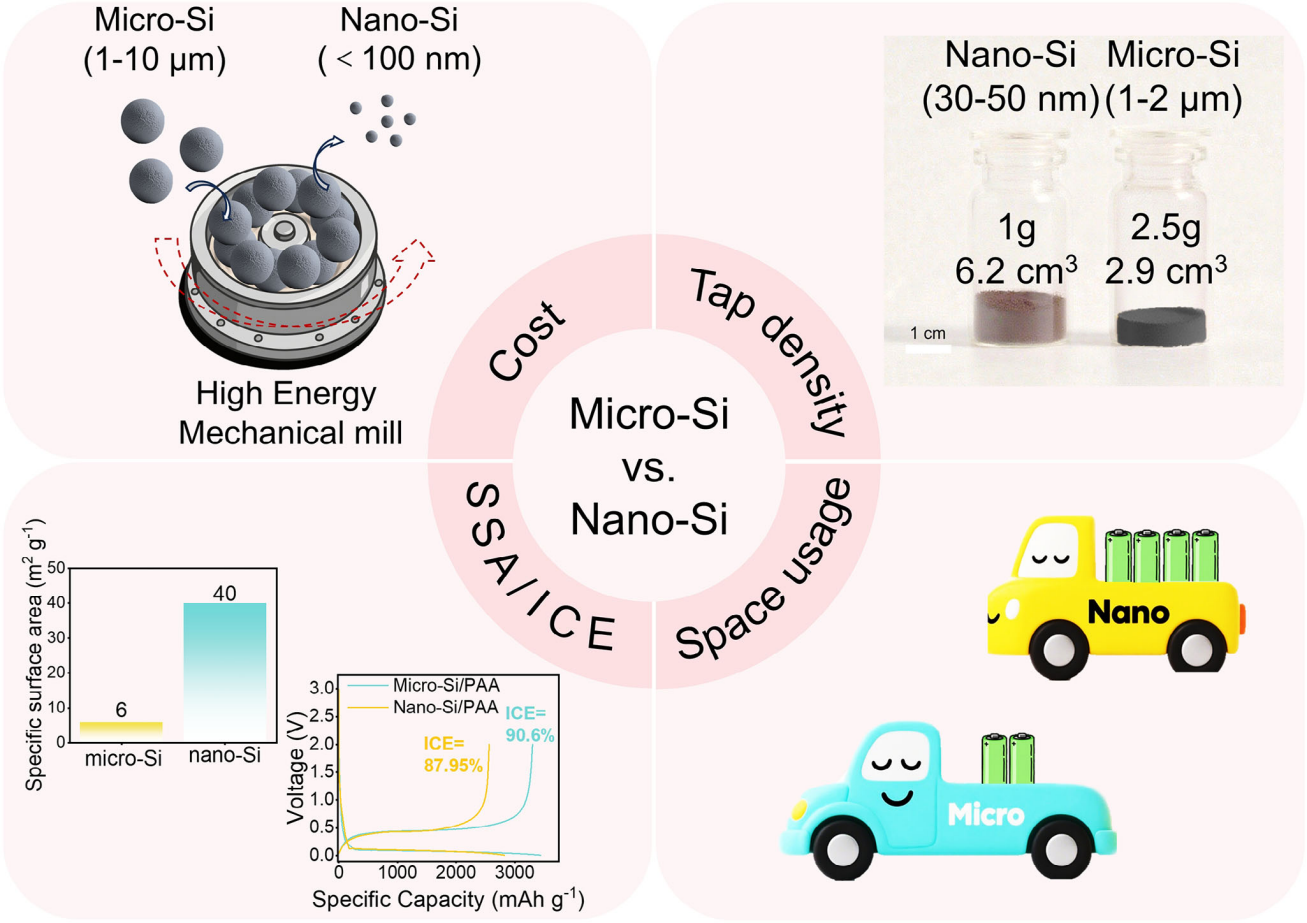
Comparison of micro‐Si and nano‐Si.

Energy shortages and severe pollution have led to a global consensus on advancing renewable energy and achieving carbon neutrality. To support these goals, advanced electrochemical energy storage systems characterized by cleanliness and high efficiency have been extensively studied.^[^ ^1^, ^2^ ^]^ Lithium‐ion batteries (LIBs) are increasingly dominant owing to their high capacity and long cycle life. However, the limited capacity of commercial graphite anodes (372 mAh g^−1^) constrains further improvements in their energy density. Consequently, silicon (Si) has emerged as a promising anode material owing to its exceptional theoretical capacity (3579 mAh g^−1^), moderate operating voltage, natural abundance, and low cost. Nevertheless, its commercialization faces considerable challenges. Its low electrical conductivity (< 10^−3^ cm^−1^) limits its rate performance. Moreover, its serious volume expansion causes structural instability during cycling.^[^ ^3^, ^4^ ^]^ Previous studies have shown that Si structures of a critical size (smaller than 150 nm)—such as nanowires, thin films,^[^ ^5^, ^6^ ^]^ and nanotubes^[^ ^7^, ^8^ ^]^ can effectively alleviate strain and prevent cracking.^[^ ^9^, ^10^ ^]^ However, the low tap density (0.16 for nano‐Si) significantly compromises volumetric energy density, which is a critical parameter for electric vehicle applications. The substantial specific surface area of nano‐Si leads to significantly lower initial Coulombic efficiency (ICE) compared to micro‐Si, primarily due to enhanced side reactions during solid electrolyte interface (SEI) formation. Additionally, energy‐intensive ball‐milling production presents substantial cost barriers to commercialization (**Figure** **1**).^[^ ^11^, ^12^, ^13^, ^14^, ^15^ ^]^

**Figure 2:**
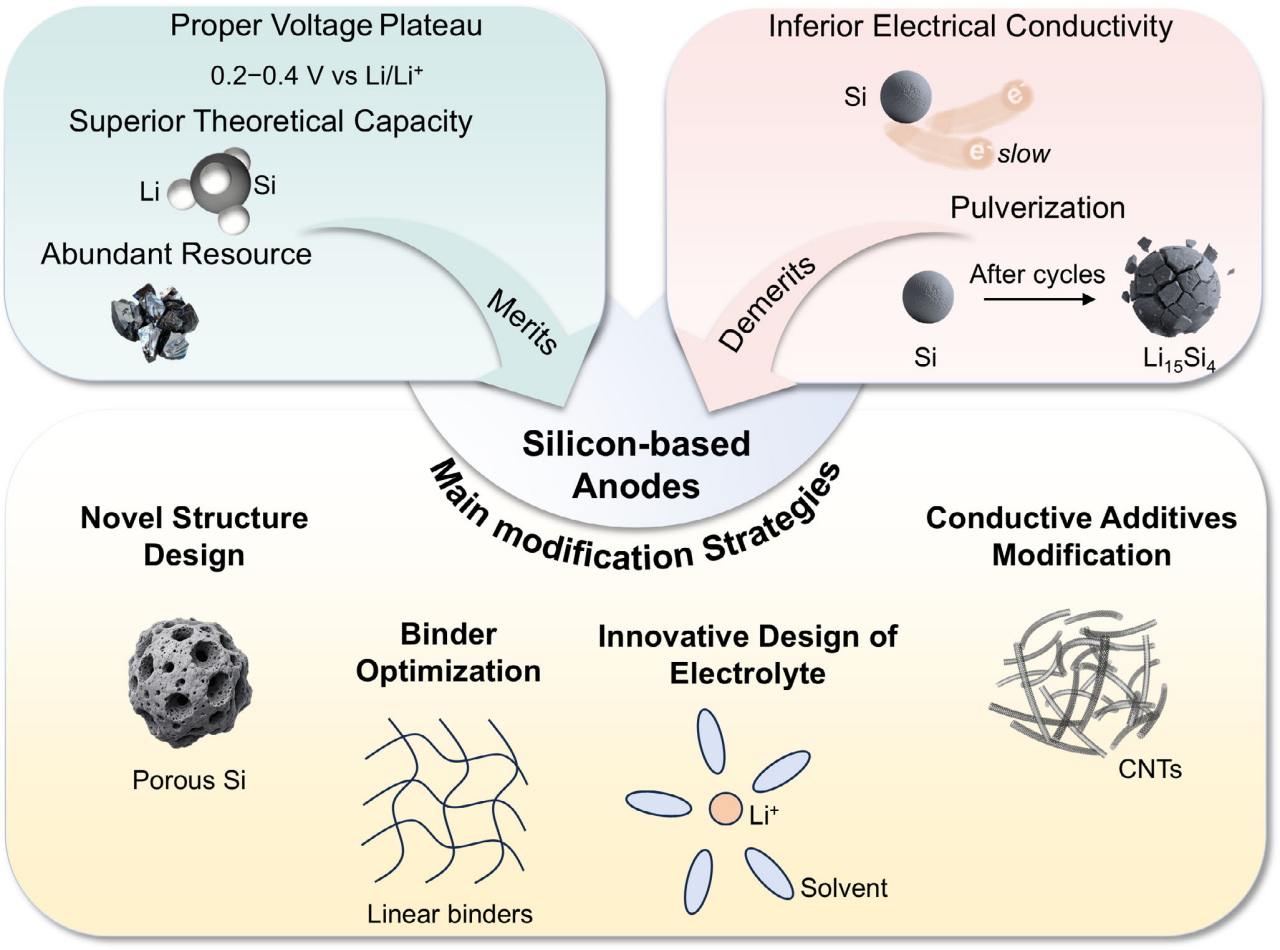
Overview of Si‐based anodes in lithium‐ion batteries.

By contrast, micro‐Si provides a higher tap density (0.86 g cm^−3^ for micro‐Si),^[^ ^16^ ^]^ reduced side reactions, and cost‐effective scalability, effectively addressing the shortcomings of nano‐Si and positioning itself as a key element of next‐generation LIBs.^[^ ^17^, ^18^, ^19^ ^]^ This review begins by examining lithium storage mechanisms and the primary challenges associated with micro‐Si anodes.^[^ ^20^ ^]^ We then methodically summarize the strategies developed to address these challenges over five years, including novel structure design, binder optimization, innovative electrolyte design, and conductive additive modification (**Figure** **2**). Moreover, we explore the potential of advanced characterization techniques and machine learning to improve material design and performance predictions in future LIBs. Lastly, we rationally consider the commercialization prospects for micro‐Si anodes.

### 1.1 Lithium Storage Mechanism of Si and Challenges of Microsized Si Anodes

Since their proposal as anode materials in 1976, Si anodes have been studied extensively.^[^ ^21^ ^]^ However, mechanical degradation and rapid capacity decay during cycling can limit their lifespan in LIBs. The corresponding electrochemical reactions are outlined in Equations (1)–(4).^[^ ^12^ ^]^

Lithiation process (*c* refers to crystalline and *a* refers to amorphous):

(1) $$Si\left(c\right)+xLi^{+}+xe^{-}\to LixSi\left(a\right)$$

(2) $$LixSi\left(a\right)+\left(3.75-x\right)Li^{+}+\left(3.75-x\right)e^{-}\to Li_{15}Si_{4}\left(c\right)$$

Delithiation process:

(3) $$Li_{15}Si_{4}\left(c\right)\to Li_{x}Si\left(a\right)+yLi^{+}+Li_{15}Si_{4}\left(c,residual\right)$$

(4) $$Li_{x}Si\left(a\right)\to Si\left(a\right)+xLi^{+}+xe^{-}$$

Upon lithiation, crystalline Si undergoes a two‐step transformation:^[^ ^22^, ^23^, ^24^ ^]^

1. At potentials above ≈0.1 V (vs Li⁺/Li), Li‐ions react with Si, forming amorphous Li_x_Si with minimal initial structural disruption;
2. When the potential drops below ≈0.1 V, amorphous Li_x_Si transforms into crystalline Li_15_Si_4_ (Equations (1) and (2)).

**Figure 3:**
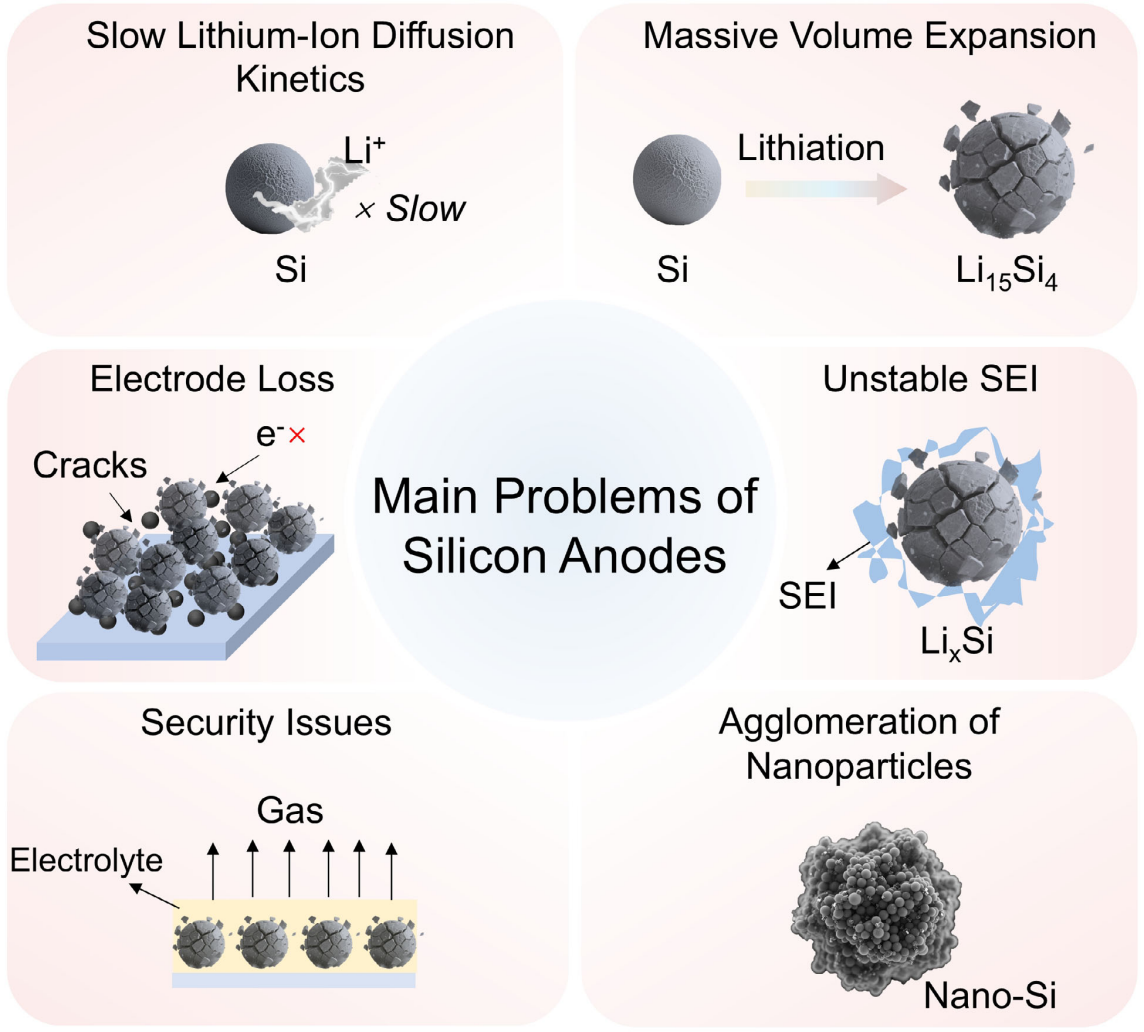
Main problems of silicon anodes.

Delithiation reverses the lithiation process, but isolated Li_15_Si_4_ persists (Equations (3) and (4)), leading to irreversible capacity loss.^[^ ^25^, ^26^, ^27^, ^28^ ^]^ In addition to the slow Li‐ions diffusion kinetics of Si itself, the most fundamental challenge comes from the ≈ 300% volume expansion during the Si phase transition. Severe internal stress causes the active material particles to break and lose electrical connection, resulting in accelerated electrode material loss and capacity attenuation. The volumetric strain also triggers a continuous SEI rupture‐reconstruction cycle, irreversibly consuming Li‐ions and increasing the interface impedance. At the same time, the decomposition of the electrolyte will produce gaseous by‐products, which will bring serious safety problems and continue to hinder the commercialization of the silicon anode.^[^ ^29^ ^]^ Although nano‐Si can effectively alleviate the mechanical stress of bulk silicon during charging and discharging by reducing its size, its high specific surface area and surface energy increase significantly, resulting in serious particle agglomeration (**Figure** **3**).^[^ ^30^, ^31^, ^32^, ^33^, ^34^ ^]^

Beyond these inherent challenges of Si, the larger particle sizes of micro‐Si exacerbate electrochemical instability, introducing unique issues detailed below.

#### 1.1.1 Increased Lithium Diffusion Path

Compared to nanoparticles, micro‐Si particles necessitate longer lithium diffusion paths during (de)lithiation, significantly increasing ionic resistance and voltage polarization. Additionally, the low intrinsic conductivity of Si impedes electron transfer, leading to elevated electrode impedance. These factors contribute to sluggish kinetics, compromising capacity retention, cycling stability, and charge‐discharge efficiency.

#### 1.1.2 Larger Local Stress Leads to Severe Particle Crushing

Volume expansion in micro‐Si generates considerable local stress, causing repeated fracturing and reducing the particle size below a critical threshold (≈150 nm).^[^ ^35^ ^]^ Without effective mitigation, this results in active material detachment, electrical disconnection, and continuous SEI growth, degrading cycle performance and stability. Therefore, the advantages of micro‐Si materials in terms of high‐volume energy density and low surface reactivity are weakened, which leads to accelerated degradation of their electrochemical performance.

## 2 Strategies for Stabilizing Micro‐Si based Negative Electrodes

Maintaining the structural stability of the electrode during the cycling process is crucial for promoting its practical application.^[^ ^36^ ^]^ Consequently, considerable effort has been made to improve the cycling performance of micro‐Si based anodes. This section presents recent developments in micro‐Si electrode design to guide future research.

### 2.1 Structural Design

#### 2.1.1 Porous Micro‐Si Structures

Over the past decade, structural engineering^[^ ^37^, ^38^ ^]^ of micro‐Si anodes has become increasingly important for addressing their inherent challenges, with porous micro‐Si emerging as a promising approach. Its abundant pore structure can buffer the stress induced by lithiation, provide space for volume expansion, enhance electrolyte infiltration, and improve lithium‐ion transport efficiency, thereby boosting its rate performance.^[^ ^39^ ^]^

**Figure 4:**
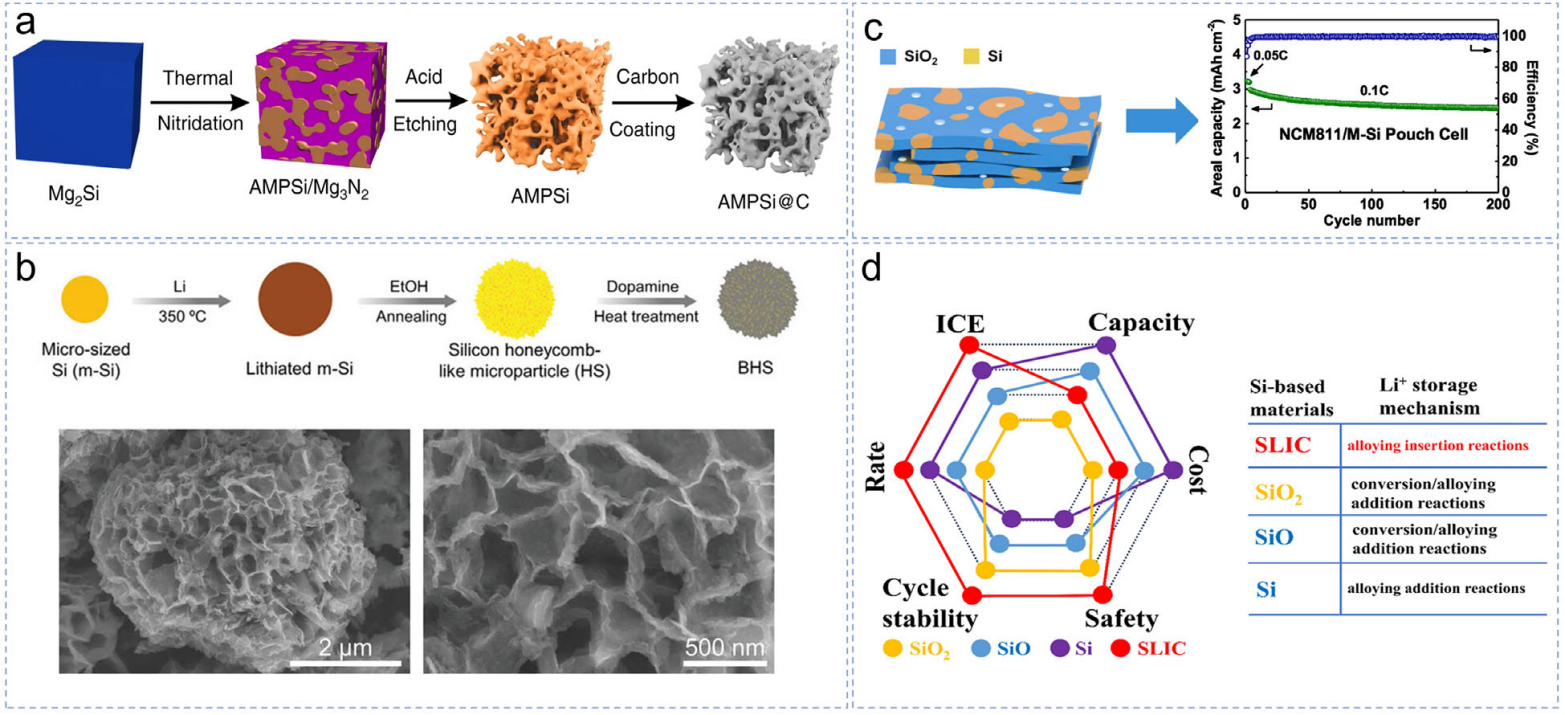
a. Schematic of the preparation of AMPSi and AMPSi@C. Reproduced with permission.^[^ ^49^ ^]^ Copyright 2019, Springer Nature. b. The fabrication process of BHS and its SEM images. Reproduced with permission.^[^ ^50^ ^]^ Copyright 2023, Wiley‐VCH. c. Schematic of montmorillonite, montmorillonite‐derived Si (M‐Si), and lithiated M‐Si. Reproduced with permission.^[^ ^52^ ^]^ Copyright 2024, American Chemical Society. d. Characteristics of the designed zero‐strain anode. Reproduced with permission.^[^ ^55^ ^]^ Copyright 2023, WILEY‐VCH.

Common methods for preparing porous Si include magnesium thermal reduction,^[^ ^40^, ^41^, ^42^, ^43^ ^]^ metal‐assisted chemical etching,^[^ ^44^, ^45^ ^]^ and dealloying of Si metal alloys.^[^ ^46^ ^]^ Additionally, porous structures can be derived directly from Si wafers or bulk materials. Bang et al. synthesized 3D macroporous Si by depositing Ag nanoparticles onto bulk Si via electrochemical reaction, followed by hydrofluoric acid (HF) etching to form porous particles.^[^ ^19^ ^]^ Another effective method is dealloying Si‐based metal alloys, which involves selectively etching specific metals to create a continuous porous network with a 3D interconnected ligament structure.^[^ ^18^, ^47^, ^48^ ^]^ An et al. produced ant‐nest‐like microporous Si (AMPSi) comprising 3D interconnected Si nanoligaments and bicontinuous nanopores through thermal nitridation of an Mg–Si alloy in nitrogen, followed by removal of the Mg_3_N_2_ by‐product in an acidic solution (**Figure** **4**a).^[^ ^49^ ^]^ This unique structure achieved an area capacity of up to 5.1 mAh cm^−2^ at a high mass loading of 2.9 mg cm^−2^. However, irregular pore formation could lead to anisotropic expansion during cycling, potentially disrupting ion transport and reducing stability. To address this problem, Wang et al. proposed a controlled isotropic canalization of micro‐Si. They employed a dual‐mechanism strategy—that is, establishing isotropic porosity through two‐dimensionally arranged Si nanosheets in radial patterns, followed by covalent/ionic bonding‐mediated consolidation of the canal structures with carbon components. This method optimized the mechanical integrity and performance and offered profound insights into the rational design and scalable fabrication of Si‐based anodes (Figure 4b).^[^ ^50^ ^]^

#### 2.1.2 Innovative Si‐Based Anodes Modification

In addition to porous designs, 2D layered structures have gained increasing attention for micro‐Si anodes.^[^ ^51^ ^]^ Chen et al. synthesized monolithic layered Si via controllable magnesiothermic reduction of montmorillonite,^[^ ^52^ ^]^ using its amorphous [SiO_4_]^4−^ framework to preserve its structural integrity (Figure 4c). A pouch cell assembled using a commercial NCM811 cathode delivered a high energy density of 655 Wh·kg^−1^ and retained 82% of its capacity after 200 cycles. To further enhance the structural integrity during cycling, Lei et al. prepared microsized Si‐based high‐entropy alloys (HEAs) by ball milling a multi‐component mixture of Cr, Mn, Fe, Co, Ni, Si, and Ge, evaluating their performance as anode materials for LIBs^[^ ^53^ ^]^. These Si‐based high‐entropy anodes exhibited enhanced cycling stability and demonstrated the potential of high‐entropy strategies for designing high‐energy‐density anodes.

Despite such advances, volume expansion remains a major problem, as commercial standards demand vertical expansion rates below 7%, which these designs have yet to consistently achieve.^[^ ^54^ ^]^ Consequently, intrinsic zero‐strain structures have garnered considerable interest. Zhao et al. developed a SiO_2_ coordinate structure with a large intra‐crystalline cavity (SLIC), formed via strong Si–O bonds, and realized inherent zero‐strain behavior in Si‐based negative electrodes.^[^ ^55^ ^]^ When fully lithiated, the coordination structure exhibited negligible disorder, demonstrating excellent cycle stability, and pioneering the design of zero‐strain structures (Figure 4d). This work reports the highest gravimetric energy density among all known zero‐/quasi‐zero‐strain anodes and provides a design framework for next‐generation zero‐strain electrodes. However, numerous structural modifications can compromise their manufacturability and cost‐effectiveness. Consequently, Cui et al. proposed a sustainable alternative by recovering micro‐Si from photovoltaic waste and using it as an anode material. This approach achieved an average coulombic efficiency of 99.94% and retained 83.13% of its initial capacity after 200 cycles, paving the way for reliable and efficient energy storage solutions.^[^ ^56^ ^]^ Beyond the structural and interfacial modifications discussed, operational strategies such as state of charge (SOC) control have emerged as an effective approach to alleviate mechanical degradation in micro‐Si anodes.^[^ ^57^, ^58^, ^59^, ^60^, ^61^ ^]^ Su et al. evaluated the differentiated electrochemical behavior of Si anodes through different voltage window definition and capacity control approaches.^[^ ^62^ ^]^ These results demonstrated that sustaining elevated lithiation states (high SOC ranges) during cycling effectively maintains low impedance in silicon anodes, thereby achieving superior cycling stability. These findings offer critical operational guidelines for maximizing the electrochemical performance of Si‐based anodes in LIBs systems.

#### 2.1.3 Si/C: Coatings and Composites

**Figure 5:**
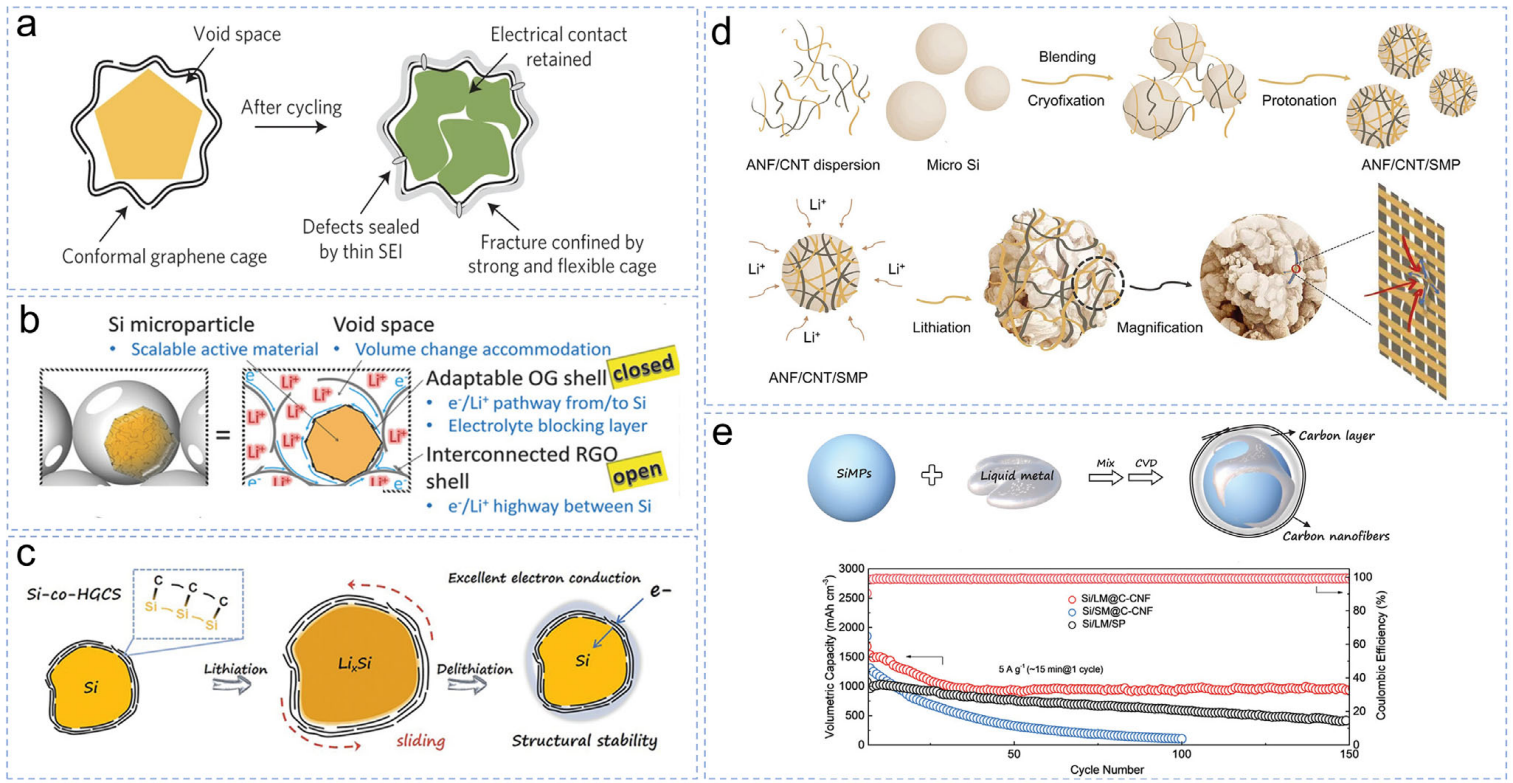
a)The operational mechanism of graphene cages. Reproduced with permission.^[^ ^68^ ^]^ Copyright 2016, Springer Nature Limited. b) Schematic of the structure of mSi@OG@RGO. Reproduced with permission.^[^ ^69^ ^]^ Copyright 2018, WILEY‐VCH. c) The covalent bonding between the Si and carbon of the SiMP (Si‐co‐HGCS). Reproduced with permission.^[^ ^70^ ^]^ Copyright 2023, Wiley‐VCH GmbH. d) The preparation process of the ANF/CNT/SiMP and the simulation diagram of the ANF/CNT network. Reproduced with permission.^[^ ^80^ ^]^ Copyright 2024, Wiley‐VCH. e) Schematic of the fabrication of Si/LM@C‐CNF. Reproduced with permission.^[^ ^86^ ^]^ Copyright 2022, Wiley‐VCH.

Carbon materials have been widely used in Si‐based anodes to improve the overall conductivity of electrodes owing to their excellent conductivity.^[^ ^63^, ^64^, ^65^, ^66^, ^67^ ^]^ The carbon coating serves a dual purpose in enhancing the performance of Si‐based anodes. First, it acts as an efficient channel for transmitting Li‐ions and electrons, improving overall conductivity. Second, it forms a protective barrier that isolates Si particles from direct contact with the electrolyte, reducing electrolyte decomposition considerably owing to interfacial side reactions and enhancing the electrochemical cycling stability of Si‐based anode materials.^[^ ^16^ ^]^ In 2016, Li et al. pioneered a highly conformal graphene cage for micro‐Si (Figure 5a),^[^ ^68^ ^]^ enabling interlayer gliding to accommodate lithiation without fracturing the Si core, stabilizing the SEI. Combined with a traditional LiCoO_2_ cathode, the graphene‐encapsulated micro‐Si demonstrated stable cycling performance, retaining 90% of its capacity after 100 cycles. However, the voids within the graphene shell could result in the loss of electrical contact owing to the large interstitial spaces and the anisotropic expansion of the Si anode during lithiation.^[^ ^69^ ^]^ To address this problem, Zhi et al. developed a novel approach by coating each Si particle with an adaptive overlapping graphene oxide (GO) sealed shell, with the exterior being formed by interconnected reduced GO (RGO), forming an open hollow structure (Figure 5b). The external continuous RGO network formed a robust conductive network, accommodating the volume expansion of single‐layer GO‐coated microcrystalline Si and facilitating rapid electron transport throughout the electrode without additional conductive additives. Although such hollow structures can enhance the structural integrity at the expense of reducing the volumetric energy density, alternative coating strategies have been explored to improve the mechanical stability under high loading. Li et al. established a covalent coating structure between micro‐Si particles and carbon shells using scalable catalytic chemical vapor deposition (Figure 5c).^[^ ^70^ ^]^ The flexible covalent Si‐C bonds created a strong link between the expanding Si particles and sliding graphene layers. This design effectively accommodated major volume changes during the charging/discharging process, absorbing mechanical stress while maintaining the anode's structural stability. Benefiting from long‐lasting electrical connection and mechanical toughness, it achieved an area capacity of up to 5.6 mAh cm^−2^ and a volume capacity of 2564 mAh cm^−3^ at high loads. This interfacial bonding effect emphasizes the stability of the coating strategy, which aims to reduce the degradation and deformation of high‐capacity materials.

To further address the mechanical challenges of micro‐Si anodes, the rational design of Si/C composites has become essential. Various carbon materials—such as graphite,^[^ ^71^, ^72^, ^73^ ^]^ carbon nanotubes (CNTs),^[^ ^74^, ^75^ ^]^ and graphene,^[^ ^76^, ^77^ ^]^ have been shown to enhance the cycle stability of Si anodes enormously. Zhang et al. designed a free‐standing Si/C film anode,^[^ ^78^ ^]^ embedding micro‐Si in a CNT network stabilized by a carbon coating to improve its durability and performance. This Si@CNT@C structure sustained electrical contact during extended cycling, with the coating bolstering mechanical and electrical properties. Liu et al. developed a new 3D network structure based on aramid nanofibers (ANFs). The team successfully achieved a uniform coating of micro‐Si particles through the synergistic effect of freeze fixation and acid‐induced protonation.^[^ ^79^ ^]^ This rice‐dumpling‐like packaging structure effectively mitigated the volume expansion of Si during lithiation. However, the poor conductivity of ANFs limited the overall electrode performance. To overcome this problem, Liu et al. applied a composite coating of ANFs and CNTs to improve the electrode conductivity (Figure 5d).^[^ ^80^ ^]^ Here, ANFs were protonated in situ on the surface of Si particles and functioned as surfactants to promote the binding of the CNTs, eventually forming an ANF/CNT network structure on the micro‐Si.

Nonetheless, these strategies struggled to prevent electrical disconnection from Si fragment detachment during repeated lithiation/delithiation. Hybridization using other materials‐such as metals,^[^ ^81^, ^82^, ^83^ ^]^ metal oxides,^[^ ^84^ ^]^ and organic polymers,^[^ ^85^ ^]^‐offers a viable solution for enhancing micro‐Si anodes’ integrality. Zhao et al. demonstrated a considerable advance by integrating liquid metal (LM) into micro‐Si, encapsulating it within a carbon shell, and growing ultralong carbon nanofibers (CNFs) in situ through LM catalysis, forming a Si/LM@C–CNF network (Figure 5e).^[^ ^86^ ^]^ This composite delivered superior mechanical and electrical properties, maintaining a capacity of 936 mAh g^−1^ after 150 cycles at a current of 5 A·g^−1^. Notably, the fluidity of LM helped to reconnect fragmented Si, effectively eliminating “dead” Si and introducing a novel paradigm for micro‐Si anode design.

#### 2.1.4 SiO_x_

Since SiO_x_ (1 ≤ x < 2) was first reported as an anode material for LIBs in the 1990s,^[^ ^87^ ^]^ its potential to enhance battery performance has garnered increasing attention owing to its high theoretical specific capacity and relatively low cost. In 2001, Tatsumisago et al. developed amorphous SiO/SnO composites with a discharge capacity of 800 mAh g^−1^.^[^ ^88^ ^]^ Subsequently, Yang et al. observed that with varying oxygen content (SiO, SiO_0.8_, and SiO_1.1_),^[^ ^89^ ^]^ the capacity decreased as the oxygen content increased. Limited Li insertion reduced the host volume expansion (less than 200%), dramatically increasing the cyclability. Moreover, the formation of Li_2_O and lithium silicates‐for example, Li_4_SiO_4_ and Li_2_Si_2_O_5_‐during initial lithiation further stabilized the structure, reducing the risk of cracking and positioning SiO_x_ as a high‐capacity, long‐life anode candidate.^[^ ^90^, ^91^, ^92^ ^]^ Despite its potential, the commercialization of SiO_x_ has been limited by its low ICE and electrochemical irreversibility.^[^ ^90^, ^93^ ^]^ This section discusses key modification strategies for SiO_x_, including surface coating, composite with other materials, elemental doping, and the chemical prelithiation of carbon‐coated and porous structure design.^[^ ^94^ ^]^

**Figure 6:**
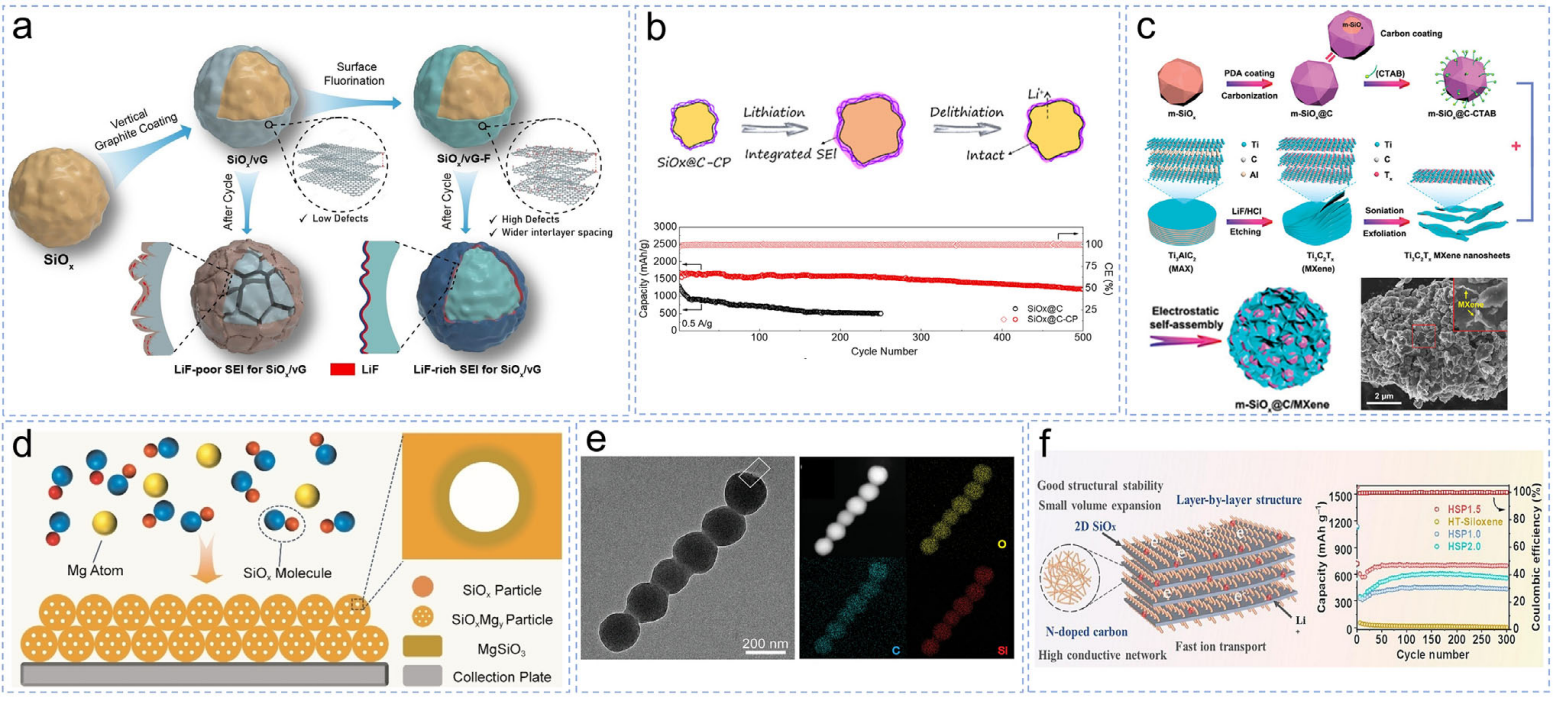
a) Schematic of the synthesis of SiO_x_@vG‐F micro‐sized particles and the protection mechanism of F‐modified vertical graphene carbon shells. Reproduced with permission.^[^ ^96^ ^]^ Copyright 2024, Wiley‐VCH. b) Schematics of CP‐integrated re‐ad‐SEI formation with π−π interaction between re‐ad‐SEI and external carbon layer. Reproduced with permission.^[^ ^97^ ^]^ Copyright 2024, American Chemical Society. c) The structural characteristics of the gradient composite d‐SiO@SiOx/C@C anode and the Finite element simulation analysis of the stress distribution of d‐SiO@SiOx/C@C after lithiation. Reproduced with permission.^[^ ^99^ ^]^ Copyright 2024, American Chemical Society. d) The schematic deposition process of SiO_x_Mg_y_. Reproduced with permission.^[^ ^102^ ^]^ Copyright 2024, Wiley‐VCH. e) TEM images of CNT@SiO*_x_*/C@C and its HAADF‐STEM and EDS element mappings. Reproduced with permission.^[^ ^107^ ^]^ Copyright 2024, Wiley‐VCH. f) Diagram of 2D SiO_x_/N‐doped carbon and the electrochemical performance. Reproduced with permission.^[^ ^108^ ^]^ Copyright 2024, Elsevier B.V.

Li et al. developed an in situ polymerization method^[^ ^95^ ^]^ to coat carbon‐encased SiO_x_ with a flexible Li‐PAA/CNT layer. The C‐SiO_x_/C anode retained a reversible capacity of 836 mAh g^−1^ after 500 cycles, demonstrating the potential of SiO_x_ particles in high‐performance anodes. To further improve the stability of the SEI, the same group developed fluorine‐doped vertical graphene (vG‐F) on carbon‐coated SiO_x_. They used the fluorine polarity to form a Li‐rich SEI, which improved the mechanical strength and ionic conductivity (**Figure** **6**a).^[^ ^96^ ^]^ Functional polymers are also frequently employed to fabricate SiO_x_ coatings. To address the fragility of traditional SEI under volume changes, Yang et al. coated carbon‐encased SiO_x_ with polyacrylonitrile (PAN), which was heat‐treated to form a conductive cyclized PAN (CP) layer (Figure 6b).^[^ ^97^ ^]^ This CP‐integrated SEI with its strong intermolecular adhesion to the carbon layer‐offered resilience and accelerated charge transport, aiding in the commercialization of high‐capacity anodes. However, the low mass loading of the SiO_x_ electrodes can limit their areal capacity. Jiang et al. addressed this drawback using a 3D large‐sheet holey graphene framework/SiO (LHGF/SiO) composite and enhanced the flexibility and conductivity via interconnected micro/nano channels.^[^ ^98^ ^]^ It redefined high‐capacity SiO_x_ design by delivering 140.8 mAh cm^−2^ at 94 mg cm^−2^ loading.

To enhance the stability of the composite structure and reduce the oxygen content in the material, Liu et al. proposed a gradient “three‐layer” SiO_x_/C composite‐micro‐SiO core, SiO_x_/C interlayer, and graphitized the carbon shell to alleviate stress and retain the structural integrity.^[^ ^99^ ^]^ Similarly, Fu et al. constructed a microsized SiO_x_ composite material (m‐SiO_x_@C/MXene) through dual confinement using carbon and MXene and dense structural engineering (Figure 6c). The results showed that m‐SiO_x_@C/MXene retained a high reversible capacity of 918 mAh g^−1^ after 400 cycles.^[^ ^100^ ^]^ Although the composite material improved the electrochemical performance, a single micro‐SiO_x_ particle was still easy to crack during the cycling process. Moreover, the synthesis process is complex, which could hinder large‐scale production. Guo et al. achieved large‐scale fabrication of magnesium‐doped SiO_x_ (SiMg_y_O_x_) microparticles using a vacuum evaporation process.^[^ ^101^ ^]^ The uniform distribution of magnesium silicate in SiMg_y_O_x_ helped to establish a bonding network inside the particles and enhanced the stability of structural evolution. Based on the SiMg_y_O_x_‐graphite anode and LiNi_0.8_Co_0.15_Al_0.05_O_2_ cathode, a 21 700 battery with a SiMg_x_O_x_‐graphite anode retained high energy density after 1000 cycles. However, it should be noted that although certain pore formation and nanostructure techniques could be expected to effectively buffer volume changes, the essential compaction density and other parameters during the actual electrode manufacturing process were often damaged. Accordingly, the same group employed an in situ Mg‐doping strategy^[^ ^102^ ^]^ to transform the closed nanoporous structure into microsized SiO_x_ particles at a high bulk density. The doped Mg atoms promoted oxygen segregation, leading to the formation of high‐density magnesium silicate. This process generated a closed nanopore structure while simultaneously increasing the tap density (Figure 6d). In order to further improve the ICE of SiO_x_ anode, Hu et al. demonstrates a synergistic modification strategy for SiO_x_ through solid‐phase Mg doping, carbon coating, and prelithiation, achieving enhanced specific capacity (1477 mAh g^−1^) while maintaining high initial Coulombic efficiency (83.77%).^[^ ^103^ ^]^

The structural design of SiO_x_ has also garnered increasing interest.^[^ ^104^, ^105^, ^106^ ^]^ Zhou et al. developed a unique chain‐like CNT@SiO_x_/C@C structure (Figure 6e),^[^ ^107^ ^]^ with CNTs as a conductive “chain” and SiO_x_ spheres as “beads,” coated with carbon to enhance the overall suppressed volume expansion. The synthesized structure maintained a capacity retention of 81.7% after 100 cycles at 0.1 A·g^−1^. The hydrothermal methods employed in the composite process could lead to the agglomeration of SiO_x_ or an incomplete carbon coating, which inevitably causes excessive local stress during the cycling process. Drawing from similar efforts with 2D architectures‐such as the Si nanosheets described before‐Fan et al. addressed these problems using small molecules to infiltrate the SiOxene structure and undergo in situ carbonization. They designed a layered structure composed of 2D SiO_x_ and nitrogen‐doped carbon and constructed a SiOxene/polyaniline (PANI) composite via in situ pyrolysis (Figure 6f).^[^ ^108^ ^]^ The carbonization of PANI provided an in situ nitrogen source and further enhanced the conductivity of the carbon framework. The material maintained a high specific capacity of 696.4 mAh g^−1^ after 300 cycles at a current density of 1.0 A·g^−1^, demonstrating its excellent performance. In addition to these strategies, binders,^[^ ^109^, ^110^, ^111^, ^112^, ^113^ ^]^ electrolytes,^[^ ^114^, ^115^, ^116^, ^117^ ^]^ and other SiO_x_ design techniques have been used to improve its electrochemical performance stability. Although SiO_x_ anodes can be viewed as the next‐generation industrial solution, many challenges must still be addressed before its commercialization.^[^ ^90^, ^91^ ^]^

Based on the systematic analysis of the above structural modification strategies, **Table** **1** further compares the latest modification performance of four typical anode systems (micro‐Si, Si/C, SiO_x_ and nano‐Si), focusing on key indicators such as ICE and cycle life. Nano‐Si has been marked with an asterisk.

**Table 1:** Summary of various structured micro‐Si, Si/C, SiO_x_, and nano‐Si based anodic LIBs.

Material	Si/SiO_x_ content [wt.%]	Capacity [mAh g^−1^]/cycles	Current density [A g^−1^]	ICE [%]	Refs.
p‐Si@g‐C_3_N_4_	99	1259/500	2	79.59	[118]
µSiG/HC@CNTs	25	750/100	0.2	88.3	[119]
µSi/G‐BC@N‐CNT	20	720/100	0.2	87.7	[120]
µSi‐PPG	1000	1913/1000	1C	92.6	[121]
µSi@MgF_2_‐1	100	1794.9/500	2.1	91.7	[122]
SiMP@C‐GN	66	1500/500	0.2	84.5	[123]
SiMP/SHP/CB	100	2617/90	0.4	>80	[124]
Si@EGaSn	10	789.5/100	0.5	90.1	[125]
sieving‐pore Si/carbon	≈90	≈2000/200	0.75	93.6	[126]
G‐Si@C	20	605/1200	1	70	[127]
Si@t‐C	85.7	1185/500	2	83.68	[128]
Si@SiC@C	–	980/800	1	88.5	[129]
Si‐co‐HGCS	96.2	1300/200	0.6	82.63	[70]
L_2_‐SiO_x_@C	28.27	654.5/500	0.5	80.4	[130]
LiF‐SiOC/C	30	≈600/100	0.4	90	[131]
*m*‐SiO_x_/C@rGO	39.6	456.9/3000	5	–	[132]
TP‐SiO/C	80	730.9/500	2	84.98	[133]
SiO*_x_*/C/MWCNTs	91.3	462/1000	1	71.2	[134]
ZnS&NC/SiO	–	748 /1000	1	–	[135]
*Si‐Ni‐HHTP	70	1405.7/100	0.1	74.41	[136]
*PANI‐Si	100	510/2000	0.5	76	[137]
*Si@FeS/C	–	669/100	0.2	≈67	[138]

### 2.2 Binder Modification

Binders play a critical role in maintaining cohesion among electrode components and ensuring a stable conductive network during the cycling process, enhancing electrochemical performance.^[^ ^139^ ^]^ For micro‐Si anodes, binders must exhibit exceptional mechanical strength and ductility to effectively mitigate the challenges associated with severe volume expansion.^[^ ^16^ ^]^

**Figure 7:**
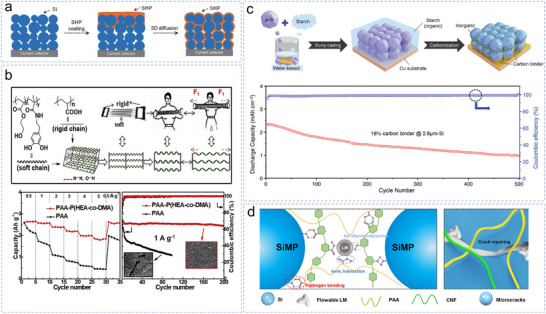
a) Schematic design of the Si–SHP/CB electrodes. Reproduced with permission.^[^ ^142^ ^]^ Copyright 2015, WILEY‐VCH Verlag GmbH & Co. KGaA, Weinheim. b) Chemical structures and illustrative interaction of P(HEA‐co‐DMA) and PAA, and the electrochemical performance of the electrode. Reproduced with permission.^[^ ^69^ ^]^ Copyright 2018, Elsevier Inc. c) Schematic of the synthesis of the carbon binder for the electrode and the cycling performance. Reproduced with permission.^[^ ^146^ ^]^ Copyright 2023, Wiley‐VCH GmbH. d) Schematic illustration of the working mechanism of Si electrodes with the PAA‐CNF‐LM binders during cycling. Reproduced with permission.^[^ ^151^ ^]^ Copyright 2023, Elsevier B.V.

Self‐healing materials, characterized by dynamic bonding networks, can repair physical damage and restore function.^[^ ^140^ ^]^ Bao et al. developed a self‐healing polymer (SHP) incorporating branched hydrogen bonds as an elastic binder for micro‐Si anodes.^[^ ^141^ ^]^ Its stretchable, self‐healing properties autonomously repair mechanical damage and cracks that occur during cycling, thereby enhancing the stability of Si particle connectivity. However, a thick binder coating on Si particles can hinder Li transport, potentially compromising battery performance. To address this, Bao et al. reconfigured the SHP into a 3D structure enveloping the Si particles by repeated scraping under heat, thereby shortening the diffusion paths and enabling faster healing (**Figure** **7**a).^[^ ^142^ ^]^ To further improve Li⁺ conductivity, polyethylene glycol (PEG) was integrated into the SHP, resulting in 80% capacity retention after 150 cycles at 0.5 C.^[^ ^143^ ^]^ Nonetheless, using organic solvents in these binders can introduce safety risks and processing challenges. For safety reasons, Xu et al. designed a water‐soluble and soft‐hard‐binding self‐healing binder,^[^ ^144^ ^]^ poly(acrylic acid)‐poly(2‐hydroxyethyl acrylate‐co‐dopamine methacrylate) (PAA‐P(HEA‐co‐DMA)) (Figure 7b), which formed a spring‐like 3D network in situ during electrode preparation that could withstand repeated deformation without structural failure and exhibited excellent rate and long cycling performance.

Unique binder design can also dramatically affect the formation, spatial distribution, and dynamic evolution of the SEI in micro‐Si anodes.^[^ ^145^ ^]^ Wang et al. developed an integrated electrode via slurry casting and carbonization, blending water‐soluble starch with micro‐Si on a copper substrate (Figure 7c).^[^ ^146^ ^]^ This electrode‐integrated structure allowed the carbon binder to form a stable and highly conductive SEI on the carbon coating. Consequently, a superior cycling stability was achieved at the current density of 2.0 mA cm^−2^ with a reversible areal capacity of 1.02 mAh cm^−2^ after 500 cycles. Conventional carbon‐based conductive agents often lose contact with Si particles amid persistent volume changes, prompting interest in conductive polymer binders that integrate binding and conductivity.^[^ ^147^, ^148^, ^149^ ^]^ Zhang et al. developed a dual‐crosslink conductive polymer network,^[^ ^150^ ^]^ which was synthesized in situ using an amidation reaction between the carboxyl groups in a high‐energy‐ dissipation polymer (PTBR) and the amino groups in SCNT‐NH_2_ during drying. PTBR combines the rigidity of PAA with the flexibility of carboxylated cyanamide rubber. It forms gradient reversible H‐bonds using highly branched tannic acid as a cross‐linking agent. This enables stress dissipation and interface stabilization through H‐bond breakage and molecular chain slippage during Si expansion.

Innovative binder designs have garnered increasing interest. Zhao et al. developed a non‐covalent assembly strategy to synthesize a liquid metal‐doped (LM‐doped) polymer binder by combining the softness of a gallium‐indium alloy with the rigidity of PAA and a cellulose nanofiber (CNF) matrix (Figure 7d).^[^ ^151^ ^]^ The resulting binder effectively dissipated the mechanical stress in the electrode through the dissociation and reconstruction of dynamic, reversible, and non‐covalent bonds. However, the relatively low capacity could limit the applicability of this method in high‐energy‐density applications. Researchers have attempted to further optimize the performance of binders using other design strategies. Ding et al. designed a layered binder^[^ ^152^ ^]^ using vacuum filtration to form a rigid GO shell on micro‐Si, topped with a soft ssDNA polymer coating via in situ self‐assembly for dual protection. The structure maintained a capacity of 1658 mAh g^−1^ after 140 cycles at a current density of 0.1 C. However, the complexity and high cost of DNA polymer synthesis can make commercialization difficult. Cui et al. synthesized an aqueous–oil binary solution‐based blend (AOB) binder inspired by spidroin.^[^ ^153^ ^]^ This binder combines high tensile strength, elasticity, and strong adhesion, stabilizing the electrode structure and extending its cycle life. Using this approach, a 3.3 Ah pouch battery with a Si/C composite anode and NCM811 cathode achieved a specific capacity of 2.92 Ah after 700 cycles. This strategy advances the commercialization potential of micro‐Si anodes enormously.

In summary, binders are essential in stabilizing micro‐Si anodes against cycling‐induced fractures, necessitating enhanced mechanical properties, interface compatibility, and functionality. Challenges such as their intricate synthesis, costly materials, and low active‐material loading demand further research to develop efficient, practical binders for micro‐Si commercialization.

### 2.3 Innovative Electrolyte Design

Severe volume expansion and particle fragmentation of micro‐Si during the cycling process can facilitate electrolyte infiltration, leading to an unstable, continuously growing SEI.^[^ ^154^ ^]^ Rational electrolyte design has become a critical focus of ongoing research, involving strategies such as the addition of functional additives,^[^ ^155^, ^156^ ^]^ optimization of high‐concentration electrolytes,^[^ ^157^, ^158^, ^159^ ^]^ and optimized Li salt to solvent ratios.^[^ ^160^, ^161^ ^]^ These measures aim to improve the structural and chemical stability of the SEI. Conventional electrolytes often form a rigid organic‐inorganic SEI on Si, which can be prone to cracking and alloy fragmentation under the stresses of volume expansion.

**Figure 8:**
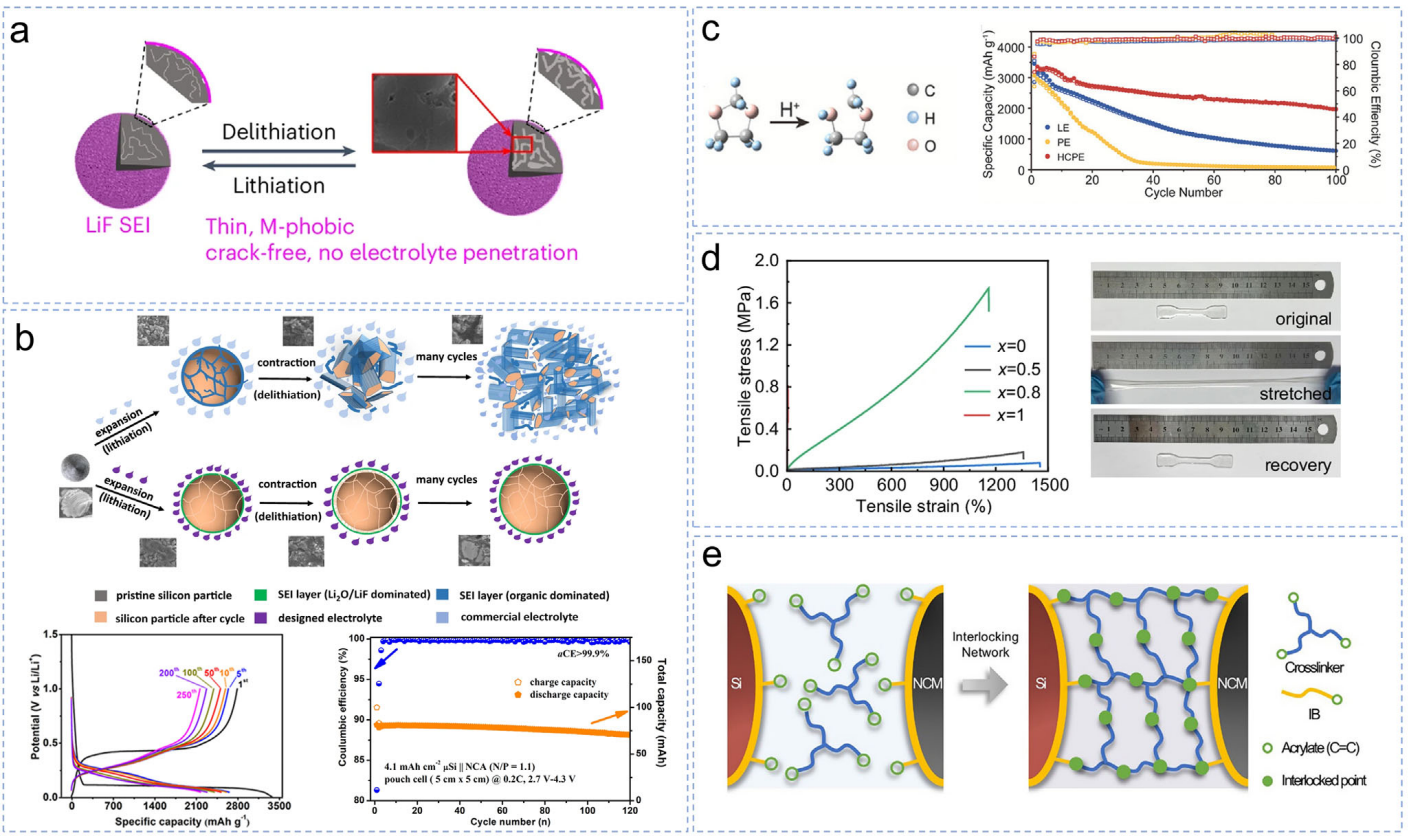
a) Stable LiF‐rich SEI maintains structural integrity during cycling. Reproduced with permission.^[^ ^174^ ^]^ Copyright 2024, Springer Nature. b) Schematic of SiMP electrodes cycled in different electrolytes and the electrochemical performance of half‐cell and pouch cell. Reproduced with permission.^[^ ^164^ ^]^ Copyright 2024, Springer Nature. c) The illustration of the ring‐opening of DOL initiated H^+^ and the cycle performance of cells assembled with different electrolytes. Reproduced with permission.^[^ ^167^ ^]^ Copyright 2023, Wiley‐VCH. d) Tensile stress‐strain curves of the elastic electrolyte with different ratios of poly‐AM and digital images of the elastic electrolyte with *x*  =  0.8. Reproduced with permission.^[^ ^172^ ^]^ Copyright 2024, Springer Nature. e) Schematic illustration of the interlocking process between electrodes and electrolytes. Reproduced with permission.^[^ ^173^ ^]^ Copyright 2025, Wiley‐VCH GmbH.

Wang et al. addressed this problem by designing a low‐adhesion SEI using 2.0 M LiPF_6_ in a 1:1 v/v THF/MTHF mixture, elevating the reduction potential above 1.1 V (versus Li/Li^+^).^[^ ^162^ ^]^ The ethers’ low reduction potential (< 0.3 V) delayed solvent decomposition until late lithiation, yielding an external LiF‐rich inner layer with minimal organic content. This enabled the micro‐Si anode (>2.5 mAh cm^−2^) to sustain a CE above 99.9% after 300 cycles. Solvent‐free ionic liquids and molecular solvent asymmetric electrolytes have been developed to minimize the organic matter content in the SEI on micro‐Si and enhance the inorganic LiF (**Figure** **8**a).^[^ ^163^ ^]^ LiPF_6_ is compatible with 1,2‐dimethoxyethane (doped with pyrrolidine cations) to form a LiF‐rich inorganic SEI on micro‐Si and other microsized alloyed anodes, providing a general solution for high‐capacity electrodes with different lithiation/delithiation potentials. The same group designed a high‐pressure electrolyte,^[^ ^164^ ^]^ forming an interface with weak bonding to a Li‐Si alloy (Figure 8b). The weak adhesion between the Si particles allowed the micro‐Si to reversibly expand and contract within the SEI shell, optimizing the capacity of the micro‐Si. The electrolyte‐assisted µSi anode achieved a Coulombic efficiency of 99.8% and capacity of 2175 mAh g^−1^ for over 250 cycle and enabled 100 mAh LiNi_0.8_Co_0.15_Al_0.05_O_2_ pouch full cells to deliver a high capacity of 172 mAh g^−1^ for 120 cycles with Coulombic efficiency of >99.9%.

Compared to traditional fluorine‐rich electrolytes, fluorine‐free electrolytes have demonstrated their effectiveness in alkaline‐ion batteries owing to their environmental friendliness and recyclability.^[^ ^165^ ^]^ Li et al. introduced a fluorine‐free 2 m LiBH_4_/THF‐MeTHF electrolyte, which could pre‐lithiate SiO_x_ on Li–Si surfaces and suppress SEI formation owing to its strong reducibility, achieving 94.3% capacity retention after 100 cycles at 0.2 C.^[^ ^166^ ^]^ To minimize excessive electrolyte consumption and the formation of a thick SEI, Wang et al. developed high‐concentration polymer electrolytes by adding LiNO_3_ as an additive to 1,3‐dioxolane, thereby inhibiting its ring‐opening polymerization reaction (Figure 8c).^[^ ^167^ ^]^ This approach formed a 2D SEI on the Si surface, effectively encapsulating individual particles and facilitating efficient Li⁺ and electron transport pathways. Although innovations in liquid electrolytes have enhanced the performance of micro‐Si anodes, their flammability under high temperatures or during overcharging can present considerable safety risks, particularly for electric vehicles.^[^ ^168^ ^]^ Solid‐state electrolytes (SSEs) are increasingly considered a safer alternative. SSEs mitigate these hazards by offering greater stability and supporting the development of Si‐based all‐solid‐state batteries. These batteries promise high capacity and an extended cycle life, making them particularly attractive for applications demanding reliable energy storage solutions.^[^ ^169^, ^170^ ^]^ Meng et al. leveraged the passivation properties of a sulfide SEI to achieve stable cycling of micro‐Si anodes.^[^ ^171^ ^]^ Unlike liquid electrolytes, SSEs limit micro‐Si contact to a 2D interface, maintaining stability throughout the operation and preventing new interface formation. Electrochemical tests revealed robust cycling life and mechanical resilience under high current densities, wide temperature ranges, and elevated areal loads. However, solid electrodes often develop internal voids and Li‐ions transport blockages owing to volume changes, typically requiring stack pressures of tens of megapascals (MPa) during fabrication. Such demands necessitate costly equipment and complex processes, which increase the production complexity and costs, creating a major bottleneck for the commercial application of SSEs. Zhou et al. addressed this problem using an elastic electrolyte synthesized via ultraviolet polymerization of dimethyl acrylamide and acrylamide in diethyl maleate (DEM), adjusting the monomer ratios to yield high ionic conductivity, elasticity, and nonflammability without external pressure (Figure 8d).^[^ ^172^ ^]^ After 300 cycles, the capacity retention rate was 90.8%, which provided a potentially new approach to SSE design. Electrolyte modification is still crucial for Si‐based anodes, and with continuous innovation, the performance and stability of the battery can be improved, thereby realizing wider application potential. In addition to solid electrolytes, gel polymer electrolytes are promising candidates for the stable operation of micro‐Si anodes. Recently, Han et al. reported the integration of gel polymer electrolytes and polymeric binders for enhancing the cycle performance of micro‐Si anodes.^[^ ^173^ ^]^ This study develops an in situ interlocking electrode‐electrolyte (IEE) system through covalent crosslinking between acrylate‐functionalized binders and quasi‐solid‐state electrolytes, effectively stabilizing interfaces in SiMP||NCM811 quasi‐solid‐state batteries suffering from large volume changes (Figure 8e).

### 2.4 Conductive Additive Modification

The intrinsic low conductivity of Si‐based anodes can limit their ability to fully exploit their high theoretical capacities. An effective strategy to enhance the overall conductivity of electrodes is the incorporation of conductive additives. Commonly used conductive additives include CNTs,^[^ ^175^, ^176^ ^]^ Super P,^[^ ^177^ ^]^ and acetylene black,^[^ ^178^ ^]^ which can improve the electrochemical cycle stability considerably by adding just 3–15 wt.%.^[^ ^179^ ^]^

**Figure 9:**
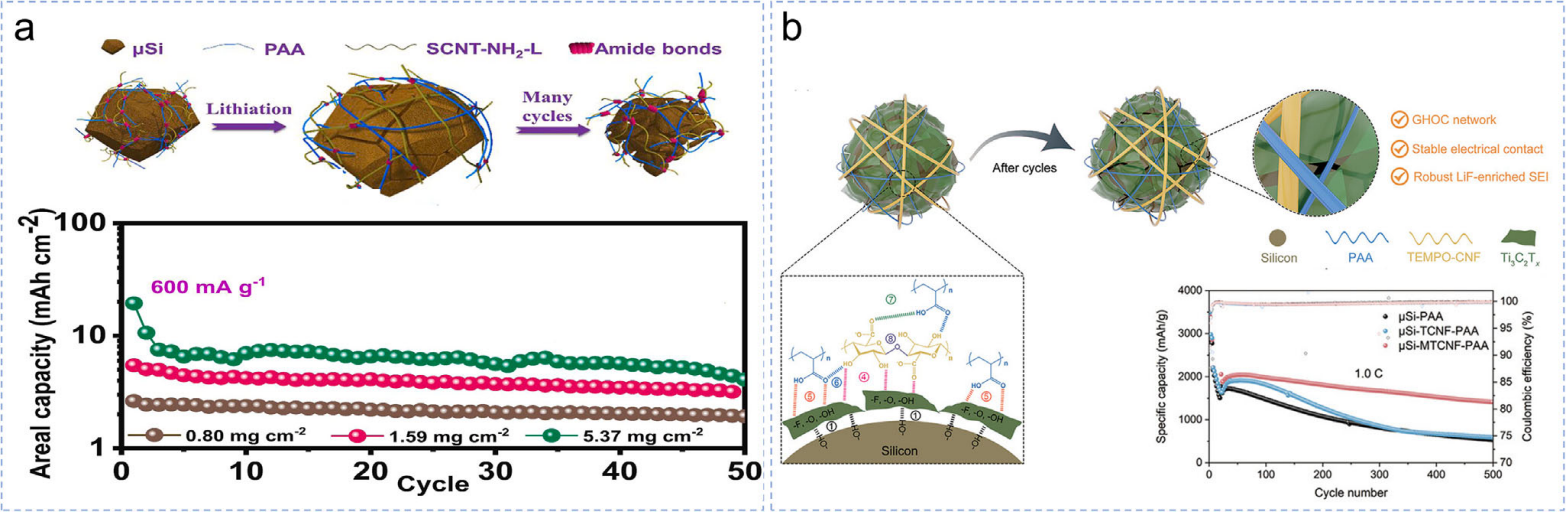
a) Schematic of the operation of the 3D conductive polymer network to buffer the volume expansion of SiMP anodes during cycles and its electrochemical performance. Reproduced with permission.^[^ ^180^ ^]^ Copyright 2022, Elsevier B.V. All rights reserved. b) µSi‐MTCNF‐PAA electrode before and after cycles and its cycling performance at 1.0 C. Reproduced with permission.^[^ ^184^ ^]^ Copyright 2024, Wiley‐VCH GmbH.

To modify CNTs to maximize the electrode capacity, Lee et al. in situ synthesized a 3D conductive polymer network.^[^ ^180^ ^]^ The carboxyl (–COOH) groups in the PAA binder were chemically bonded to the amine (–NH_2_) groups of amino‐functionalized long single‐walled carbon nanotubes (SWCNTs) during drying (with SWCNT–NH_2_‐L as a conductive additive) (**Figure** **9**a). This synergy buffers the volume expansion and sustains electrical contact, achieving 10.59 mAh cm^−2^ at 5.37 mg cm^−2^ loading and 600 mA·g^−1^. The modified CNTs obtained by simple chemical oxidation treatment maintained their conductivity and could be easily dispersed in water without additional treatment.^[^ ^181^ ^]^ Kim et al. obtained large‐scale oxidized‐controlled CNT scaffolds with high conductivity through mild surface treatment.^[^ ^182^ ^]^ The self‐assembled interwoven network, micro‐Si particles, and the polymer binder formed a stable aqueous slurry. The rheological analysis confirmed its scalability, enabling 30 × 40 cm electrodes with industrial potential. Beyond CNTs, other conductive media have also garnered attention. Shi et al. synthesized hollow graphite carbon (HGC) shells of both micro and nanometer sizes^[^ ^183^ ^]^ and used them as conductive additives for micro‐Si anodes to regulate the strain and enhance the kinetic processes. The hollow structure provided space for volume expansion and effectively mitigated particle fragmentation. Finally, an impressive capacity of 651.4 mAh g^−1^ was obtained after 500 cycles at a high current density of 2 A·g^−1^. However, the HGC preparation process can be overly complex for commercialization purposes.

Ma et al. designed a gradient hierarchically ordered conductive network structure in which flexible Ti_3_C_2_T_x_ nanosheets tightly encapsulated micro‐Si particles to form a highly conductive inner layer.^[^ ^184^ ^]^ A porous reinforced outer layer was constructed by self‐assembly of oxidized cellulose nanofibers (TCNFs) and polyacrylic acid (PAA) on the surface of Ti_3_C_2_T_x_‐coated micro‐Si. The composite structure realized the close interconnection between the components through the synergistic effect of covalent and hydrogen bonds. The anode exhibited a high discharge capacity of 1413.7 mAh g^−1^ after 500 cycles at 1.0 C, providing a simple method for stable bulk‐phase and interphase network development (Figure 9b).

## 3 Advanced Characterization Techniques

Macro‐electrochemical tests (including cycle stability, rate performance, and cyclic voltammetry analysis) are important in evaluating electrode performance. However, unraveling the material structure and performance requires more advanced techniques such as transmission electron microscopy (TEM), scanning electron microscopy (SEM), and Fourier‐transform infrared (FTIR) spectroscopy.^[^ ^185^ ^]^ These methods can reveal the relationship between the electrode structure and electrochemical behavior, offering a scientific foundation for optimizing performance. However, in the ex situ characterization process, the exposure of a sample to an oxygen‐rich/water‐containing environment can lead to contamination and affect the accuracy of the test results owing to changes in the reaction conditions. By contrast, in situ characterization techniques can mitigate these limitations. This section systematically reviews three in situ characterization techniques, namely optical, electrical and mechanical methods, and their latest research progress. It aims to provide researchers with a comprehensive research method for battery materials at the atomic/electronic scale.

### 3.1 Optical Characterization

Raman spectroscopy is a scattering technique that provides information on molecular vibrations (including lattice vibrations) and rotational energy levels based on the spectra of scattered light measured under varying incident light frequencies. It is widely used in chemistry, materials science, and physics owing to its rich informational output and simple sample preparation requirements.^[^ ^186^ ^]^ However, with the increasing complexity in research systems, the limitations of traditional Raman spectroscopy have become more apparent. Nonetheless, in LIBs, Raman spectroscopy exhibits high sensitivity to structural changes in the crystalline materials and can be employed to monitor alterations during the electrochemical cycling process. In situ Raman electrochemistry, that is, the combination of Raman spectroscopy and electrochemical testing systems, can enable real‐time battery monitoring of the charging and discharging processes and offer direct observation of molecular structures and dynamic transformations.^[^ ^187^ ^]^

**Figure 10:**
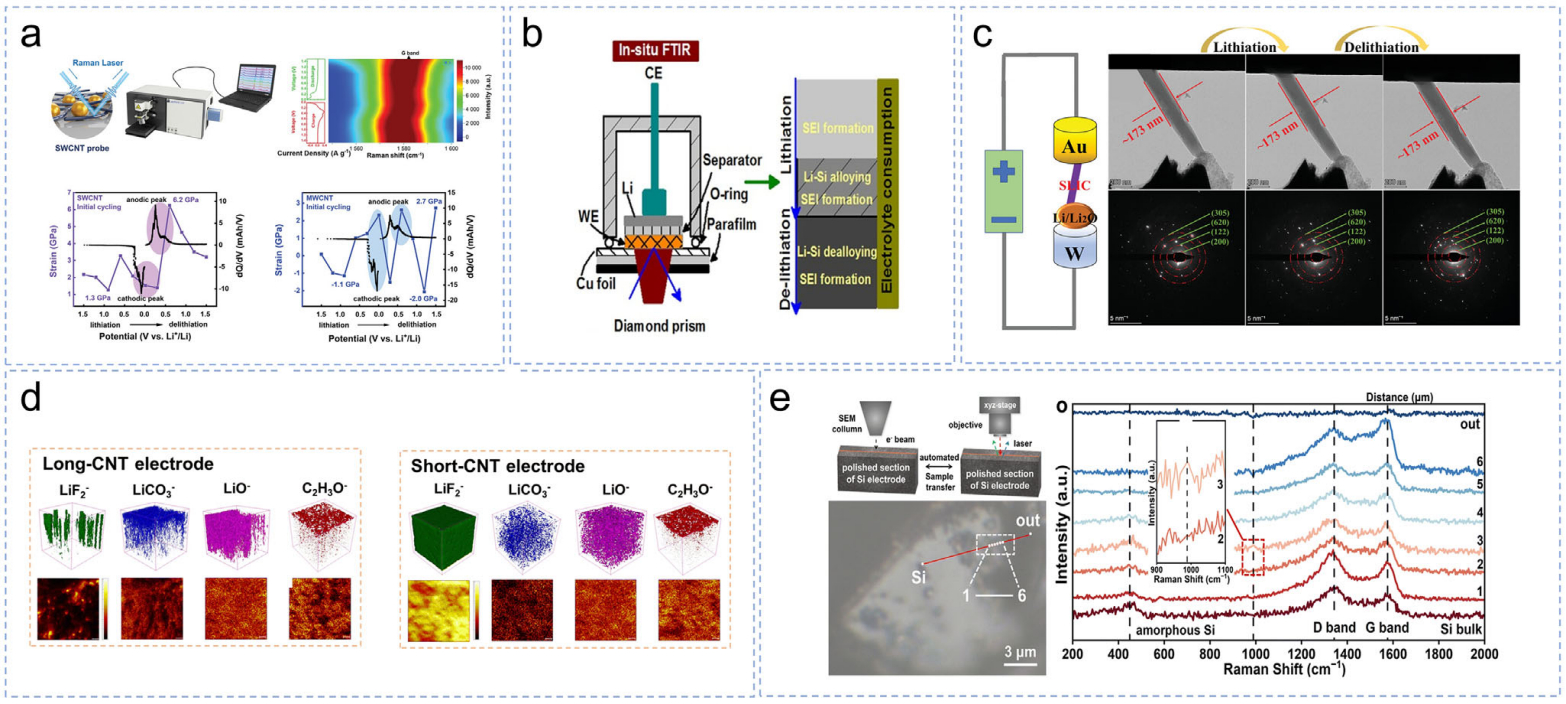
a) Stress analysis in SWCNTs and MWCNTs during cycling under in situ Raman spectroscopy. Reproduced with permission.^[^ ^176^ ^]^ Copyright 2023, Wiley‐VCH GmbH. b) Diagram of the in situ FTIR working principle, reproduced with permission.^[^ ^188^ ^]^ Copyright 2016, WILEY‐VCH Verlag GmbH & Co. KGaA, Weinheim. c)Illustration of the in situ TEM testing setup, showing the TEM morphology and selected electron diffraction images of the SLIC anode during cycling. Reproduced with permission.^[^ ^55^ ^]^ Copyright 2023, Wiley‐VCH GmbH. d) Cryo‐TEM analysis of the SEI composition for the long‐CNT electrodes and the short‐CNT electrodes. Reproduced with permission.^[^ ^175^ ^]^ Copyright 2024, the Royal Society of Chemistry. e) SEM‐Raman analysis a Si particle. Reproduced with permission.^[^ ^189^ ^]^ Copyright 2024, the Royal Society of Chemistry.

Wang et al. adopted a novel approach by not restricting the fragmentation of Si‐based particles and used in situ Raman spectroscopy alongside density functional theory (DFT) calculations to reveal the mechanism behind the enhanced performance of Si‐based anodes modified with SWCNTs. Their study revealed that SWCNTs use volume expansion to induce interfacial reactions during the cycling process, stabilizing fragmented Si clusters in situ.^[^ ^74^ ^]^ This provides valuable insight into CNT modification strategies and their impact on the performance and stability of Si‐based anodes. He et al. conducted in situ Raman experiments to monitor the stress evolution within CNTs in a SiO_x_@C anode during cycling (**Figure** **10**a).^[^ ^176^ ^]^ By analyzing the shift of the G‐band, they inferred strain variations in the CNTs and speculated on the volume expansion of the SiO_x_@C material and its interaction with CNTs. Compared to Raman spectroscopy, infrared spectroscopy exhibits higher light absorption intensity owing to the molecular vibration modes, enhancing its sensitivity in detecting low‐concentration samples. This makes IR spectroscopy particularly useful for component identification and analysis. In situ FTIR spectroscopy can monitor real‐time chemical and compositional changes through infrared absorption associated with molecular vibrations. Yang et al. used an advanced in situ FTIR‐ATR spectro‐electrochemical cell to observe the electrode/electrolyte interfacial reactionships in the Si anodes of LIBs (Figure 10b).^[^ ^188^ ^]^ Their findings indicated that SEI formation predominantly occurred during delithiation, providing new insights into the evolution and degradation of SEI.

### 3.2 Electronic Characterization

Photoelectronic characterization methods can obtain rich information on the surface, interface, and bulk structure of materials by detecting the interaction between materials and light, and they have become an indispensable tool for material science and surface chemistry research. At present, advanced characterization techniques represented by in situ TEM and time‐of‐flight secondary ion mass spectrometry (TOF‐SIMS) play a key role in the study of electrochemical mechanisms and related processes. Wang et al. employed in situ TEM to study the phase transitions and lattice parameter changes of a synthesized zero‐strain SLIC material during the cycling process. They confirmed the zero‐strain behavior of the SLIC (Figure 10c), which demonstrated no change in morphology during the charge/discharge cycles, maintaining a stable diameter of 173 nm. Both the morphology and phase structure remained unaffected across the different lithiation states. These results highlighted the exceptional stability of the SLIC electrode, with no changes in the size, phase, or lattice parameters during the cycling process.

TOF‐SIMS is a high‐resolution surface analysis technique primarily used to investigate the elemental composition, molecular structure, chemical states, and kinetic processes of surface chemical reactions. In recent years, the technique has played a crucial role in characterizing the compositions and morphologies of the SEI. He et al. used TOF‐SIMS to characterize the SEI composition of electrodes containing long and short nanotubes, respectively.^[^ ^175^ ^]^ The SEI of an electrode comprising long CNTs had fewer LiF_2_^−^fragments than that of an electrode comprising short CNTs (Figure 10d). Moreover, the inorganic components of the SEI were further validated by cryo‐TEM, which revealed a greater presence of crystalline LiF lattices in the SEI of the short‐CNT‐composed electrode. These spectroscopic results confirmed that the short‐CNT‐composed electrode had a LiF‐rich SEI. Complementing these surface‐sensitive techniques, Wang et al. combined Raman spectroscopy with SEM,^[^ ^189^ ^]^ which enabled simultaneous imaging and detection at the same location (Figure 10e), confirming the enhanced conductivity of the SEI.

### 3.3 Mechanical Characterization

**Figure 11:**
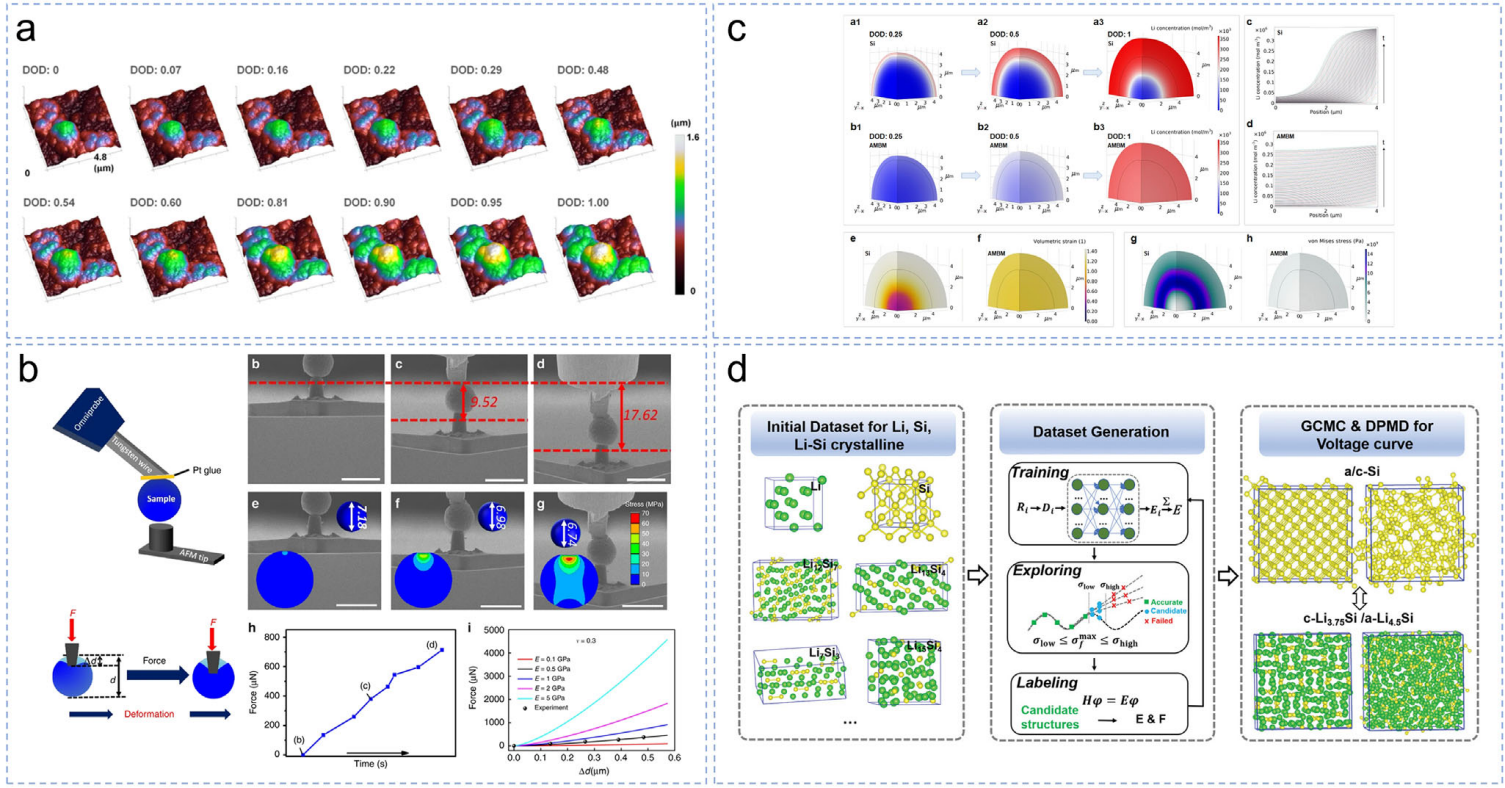
a) Morphological evolution of anodes. Reproduced with permission.^[^ ^190^ ^]^ Copyright 2023, Springer Nature. b) Schematic of the in situ AFM–SEM experiment for measuring mechanical strength. Reproduced with permission.^[^ ^191^ ^]^ Copyright 2020, Springer Nature. c) Finite element simulations of Si and Si_8.5_Sn_0.5_Sb‐AMBM anodes at different lithiation depths. Reproduced with permission.^[^ ^190^ ^]^ Copyright 2023, Springer Nature. d) Workflow for constructing the deep potential model using the deep potential generator (DP‐GEN) and applying the DP model to simulate the electrochemical property evolution during lithiation/delithiation. The green and yellow balls represent Li and Si atoms, respectively. Reproduced with permission.^[^ ^98^ ^]^ Copyright 2023, Wiley‐VCH.

Atomic force microscopy (AFM) is an important tool in LIB research. It captures the microscopic morphology of the electrode surface in real time through contact between the probe and the electrode atom. It provides physicochemical information at the nanoscale level, allowing experimental insights into optimizing electrode materials and electrolytes. Zhang et al. visually examined the morphological changes in anode particles during the cycling process using an in situ AFM test (**Figure** **11**a).^[^ ^190^ ^]^ This study demonstrated that controlling the concentration of Si combined with Sn and Sb through arc melting and high‐energy ball milling enhanced the mechanical stability of Si‐rich anodes at the micrometer scale during the cycling process. However, despite its high resolution, AFM has a slow imaging speed, which makes advanced elemental analysis and large‐area characterization challenging. By contrast, SEM enables elemental analysis by capturing the sample surface over a wide area while maintaining excellent resolution. Consequently, in situ AFM‐SEM is a valuable characterization method that leverages the benefits of both approaches while minimizing their respective disadvantages. Consequently, Zhang et al. used an in situ AFM–SEM method to determine the mechanical strength of CNT@Si@C (Figure 11b).^[^ ^191^ ^]^ Outstanding mechanical strength was demonstrated by CNT@Si@C, which could endure pressures of up to 200 MPa or more without breaking or deforming.

### 3.4 Finite Element Analysis and Machine Learning

Compared with traditional experimental methods, numerical simulation techniques can be used to theoretically reveal and evaluate microscopic processes that are difficult to observe directly, thereby providing critical mechanistic insights and theoretical support for experimental phenomena.^[^ ^192^ ^]^ In recent years, numerical simulation has garnered considerable attention in the study of diffusion‐induced stress in electrode materials and has become an important research technique for understanding the mechanisms of stress evolution. Zhang et al. used finite element method simulations to study the Li concentration distribution and stress state of individual anode particles during the lithiation of Si_8.5_Sn_0.5_Sb‐AMBM (Arc melting‐ball milling) and Si anodes (Figure 11c).^[^ ^190^ ^]^ Lithiation started at the anode surface and moved inside during the discharge cycle, causing variable volumetric and stress changes. Lithiation of the Si anode required more time to reach its center, which resulted in a clear phase boundary between the Li‐rich and Li‐poor regions. By contrast, the Si_8.5_Sn_0.5_Sb‐AMBM anode exhibited a more uniform lithiation expansion, leading to lower von Mises stress levels.

Machine learning (ML) is an emerging technology that offers enormous potential for discovering new materials, optimizing electrochemical processes, and predicting battery life in the field of LIBs,^[^ ^193^, ^194^ ^]^ considerably improving the efficiency of scientific research. The performance of high‐capacity Si anodes in LIBs and their connection to the chemical pathways and structural evolution during charging and discharging operations were examined by Fu et al. using ML techniques (Figure 11d).^[^ ^195^ ^]^ To investigate the lithiation/delithiation process of Si anodes, they trained a high‐accuracy Li–Si deep potential that covered the entire composition space. This ML‐driven approach enabled them to accurately predict the dynamic behavior of Li–Si interaction, revealing key insights into the phase transition and stress evolution. An important development in understanding the electrode materials and response mechanisms has been the combination of several characterization techniques. Zhang et al. used in situ spectroscopy (FTIR and Raman) and DFT calculations to study Li transport in poly(hexaazatrinaphthalene)‐coated (PHATN‐coated) Si/C electrodes.^[^ ^85^ ^]^ Although improved characterization methods and ML could not directly improve battery performance, they proved critical for assessing the particle shape, mechanical characteristics, and electrode/interface composition. Future characterization techniques should give researchers greater insights as technological advances and materials sciences become better understood.

## 4 Conclusion

Compared to conventional anode materials, micro‐Si offers superior advantages in terms of cost, natural availability, and voltage performance, making it a prime candidate for future battery technologies. However, problems such as their poor conductivity and size enlargement, which can lead to extended Li‐ions diffusion paths, stress concentration, and severe powder degradation must still be addressed. Advanced characterization has deepened insights into their failure mechanisms and spurred modification strategies to enhance their cycling stability. This review outlines the Si‐anode lithiation mechanisms and evaluates the commercialization prospects and hurdles of micro‐Si anodes. Recent advances in structural design, binder optimization, and electrolyte innovation for micro‐Si anodes are also detailed herein. Integrating artificial intelligence and cutting‐edge characterization techniques lays a foundation for micro‐Si optimization. Synergistic material engineering, refined electrode architectures, and advanced diagnostics herald a revival of micro‐Si anodes in high‐energy‐density LIBs.

1. The increasing industrial demand for energy storage with high volumetric and gravimetric capacities highlights the need to optimize micro‐Si tap density and suppress volume expansion. Tailored particle size and spatial distribution can mitigate local stress, bolster structural integrity, and extend lifespan. The transition from laboratory‐scale research to practical high‐energy battery applications requires more in‐depth investigation. Key measures include electrode compaction and high load, as well as expanding battery size during operation.^[^ ^196^, ^197^, ^198^ ^]^ Moreover, advanced in situ characterization techniques are essential for understanding the reaction and failure mechanisms at the interface between the micro‐Si electrodes and electrolytes. Taking advantage of existing developments and improved characterization methods could enhance electrode stability and accelerate the commercialization of micro‐Si anodes.
2. The optimal design of the binder system is crucial to improve the CE, and to maximize electrode performance by minimizing the amount of binder. Factors affecting micro‐Si electrode binder design include the following: (1) Binder system designs should achieve a functionality comparable to current industrial standards with a low binder content (<3 wt.%); (2) Single‐function binders are increasingly inadequate for micro‐Si anodes. When combined with self‐healing properties, high adhesion and mechanical strength can result in synergistic effects (1 + 1 > 2). (3) The development of binders with Li storage capacity is required to further enhance the energy density.
3. Rational electrolyte design enhances SEI stability via high ionic conductivity, flexibility, and viscosity to accommodate volume shifts. However, there is still a dearth of systematic analyses of the interactions between the electrolyte additives and micro‐Si anodes. Combining several electrolyte additives could yield better results to satisfy the increased development requirements of micro‐Si anodes. Moreover, electrolytes and additives should be thermally and electrochemically stable while maintaining a low reduction potential and solvation ability to ensure safety.^[^ ^199^ ^]^
4. ASSBs offer superior safety and high energy density, which is driving their rapid development. Integrating micro‐Si anodes with solid electrolytes can suppress SEI growth, enhance short‐circuit resistance, and improve durability and energy efficiency. However, unlike liquid‐electrolyte batteries, ASSBs struggle with maintaining interfacial connectivity and mechanical compatibility. Moreover, incompatibility between the solid electrolyte and active material can lead to space‐charge layers, side reactions, and increased impedance. The key to ASSB commercialization is preserving the structural integrity of the Si‐anode/solid electrolyte interface and interparticle junctions under volume changes and stress while minimizing interfacial side reactions.^[^ ^200^ ^]^

Although the commercialization of micro‐Si anodes for LIBs faces challenges, breakthroughs have been made through continuous research efforts. This review highlights the progress and insights from studies on micro‐Si based anodes in LIBs. With the support of cooperative research, the commercialization of Si‐based anodes should achieve enormous success.

## Conflict of Interest

The authors declare no conflict of interest.

## References

[1] J. M. Tarascon , M. Armand , Nature 2001, 414, 359. 11713543 10.1038/35104644

[2] M. Li , J. Lu , Z. Chen , K. Amine , Adv. Mater. 2018, 30, 1800561. 10.1002/adma.201800561 29904941

[3] Y. Jin , B. Zhu , Z. Lu , N. Liu , J. Zhu , Adv. Energy Mater. 2017, 7, 1700715.

[4] J.‐Y. Li , Q. Xu , G. Li , Y.‐X. Yin , L.‐J. Wan , Y.‐G. Guo , Mater. Chem. Front. 2017, 1, 1691.

[5] M. Shimizu , H. Usui , T. Suzumura , H. Sakaguchi , J. Phys. Chem. C 2015, 119, 2975.

[6] H. Usui , M. Shimizu , H. Sakaguchi , J. Power Sources 2013, 235, 29.

[7] Z. Li , M. Han , P. Yu , J. Lin , J. Yu , Nano‐Micro Lett. 2024, 16, 98. 10.1007/s40820-023-01308-x PMC10825112 38285246

[8] H. Wu , G. Chan , J. W. Choi , I. Ryu , Y. Yao , M. T. McDowell , S. W. Lee , A. Jackson , Y. Yang , L. Hu , Y. Cui , Nat. Nanotechnol. 2012, 7, 310. 22447161 10.1038/nnano.2012.35

[9] M. Shimizu , H. Usui , K. Matsumoto , T. Nokami , T. Itoh , H. Sakaguchi , J. Electrochem. Soc. 2014, 161, A1765.

[10] Z. Xu , J. Yang , H. Li , Y. Nuli , J. Wang , J. Mater. Chem. A 2019, 7, 9432.

[11] A. S. Aricò , P. Bruce , B. Scrosati , J.‐M. Tarascon , W. van Schalkwijk , Nat. Mater. 2005, 4, 366. 15867920 10.1038/nmat1368

[12] C.‐M. Park , J.‐H. Kim , H. Kim , H.‐J. Sohn , Chem. Soc. Rev. 2010, 39, 3115. 20593097 10.1039/b919877f

[13] J. W. Choi , D. Aurbach , Nat. Rev. Mater. 2016, 1, 16013.

[14] M. A. Rahman , G. Song , A. I. Bhatt , Y. C. Wong , C. Wen , Adv. Funct. Mater. 2016, 26, 647.

[15] C. Zhang , W. Lv , Y. Tao , Q.‐H. Yang , Energy Environ. Sci. 2015, 8, 1390.

[16] Z. Zhao , F. Chen , J. Han , D. Kong , S. Pan , J. Xiao , S. Wu , Q.‐H. Yang , Adv. Energy Mater. 2023, 13, 2300367.

[17] R. Yi , F. Dai , M. L. Gordin , S. Chen , D. Wang , Adv. Energy Mater. 2013, 3, 295.

[18] M. Sohn , D. G. Lee , H.‐I. Park , C. Park , J.‐H. Choi , H. Kim , Adv. Funct. Mater. 2018, 28, 1800855.

[19] B. M. Bang , J.‐I. Lee , H. Kim , J. Cho , S. Park , Adv. Energy Mater. 2012, 2, 878.

[20] W. He , W. Xu , Z. Li , Z. Hu , J. Yang , G. Qin , W. Teng , T. Zhang , W. Zhang , Z. Sun , X. Yu , Adv. Sci. 2025, 12, 2407540. 10.1002/advs.202407540 PMC11809347 39783835

[21] S. C. Lai , J. Electrochem. Soc. 1976, 123, 1196.

[22] H. Li , X. Huang , L. Chen , G. Zhou , Z. Zhang , D. Yu , Y. Jun Mo , N. Pei , Solid State Ionics 2000, 135, 181.

[23] P. Limthongkul , Y.‐I. Jang , N. J. Dudney , Y.‐M. Chiang , Acta Mater. 2003, 51, 1103.

[24] A. Netz , R. A. Huggins , W. Weppner , J. Power Sources 2003, 119‐121, 95.

[25] Y. Park , N.‐S. Choi , S. Park , S. H. Woo , S. Sim , B. Y. Jang , S. M. Oh , S. Park , J. Cho , K. T. Lee , Adv. Energy Mater. 2013, 3, 206.

[26] J. P. Maranchi , A. F. Hepp , P. N. Kumta , C. High , Electrochem. Solid‐State Lett. 2003, 6, A198.

[27] J. H. Ryu , J. W. Kim , Y.‐E. Sung , S. M. Oh , Electrochem. Solid‐State Lett. 2004, 7, A306.

[28] M. Wada , J. Yin , E. Tanabe , Y. Kitano , S. Tanase , O. Kajita , T. Sakai , Electrochemistry 2003, 71, 1064.

[29] J. E. Entwistle , S. G. Booth , D. S. Keeble , F. Ayub , M. Yan , S. A. Corr , D. J. Cumming , S. V. Patwardhan , Adv. Energy Mater. 2020, 10, 2001826.

[30] M. Kim , Z. Yang , I. Bloom , J. Electrochem. Soc. 2021, 168, 010523.

[31] Y. Liao , H. Zhang , Y. Peng , Y. Hu , J. Liang , Z. Gong , Y. Wei , Y. Yang , Adv. Energy Mater. 2024, 14, 2304295.

[32] E. Peled , S. Menkin , J. Electrochem. Soc. 2017, 164, A1703.

[33] X. Li , T. Zeng , H. Qin , R. Huo , Y. Liu , D. Wei , X. Ding , J. Energy Storage 2021, 42, 103113.

[34] S. K. Heiskanen , J. Kim , B. L. Lucht , Joule 2019, 3, 2322.

[35] X. H. Liu , L. Zhong , S. Huang , S. X. Mao , T. Zhu , J. Y. Huang , ACS Nano 2012, 6, 1522. 22217200 10.1021/nn204476h

[36] Feng , Z.‐y. , Peng , W.‐j. , Wang , Z.‐x. , Guo , H.‐j. , Li , X.‐h. , Yan , G.‐c. , J.‐x. Wang , Int. J. Miner., Metall. Mater. 2021, 28, 1549.

[37] A. Kothuru , A. Cohen , G. Daffan , Y. Juhl , F. Patolsky , Carbon Energy 2024, 10.1002/cey2.507.

[38] A. Reyes Jiménez , R. Klöpsch , R. Wagner , U. C. Rodehorst , M. Kolek , R. Nölle , M. Winter , T. Placke , ACS Nano 2017, 11, 4731. 28437078 10.1021/acsnano.7b00922

[39] W. Cao , K. Han , M. Chen , H. Ye , S. Sang , Electrochim. Acta 2019, 320, 134613.

[40] H. Jia , P. Gao , J. Yang , J. Wang , Y. Nuli , Z. Yang , Adv. Energy Mater. 2011, 1, 1036.

[41] J. Entwistle , A. Rennie , S. Patwardhan , J. Mater. Chem. A 2018, 6, 18344.

[42] Z. Bao , M. R. Weatherspoon , S. Shian , Y. Cai , P. D. Graham , S. M. Allan , G. Ahmad , M. B. Dickerson , B. C. Church , Z. Kang , H. W. Abernathy Iii , C. J. Summers , M. Liu , K. H. Sandhage , Nature 2007, 446, 172. 17344850 10.1038/nature05570

[43] L. Shi , W. Wang , A. Wang , K. Yuan , Y. Yang , J. Alloys Compd. 2016, 661, 27.

[44] T. Zhao , D. Zhu , W. Li , A. Li , J. Zhang , J. Power Sources 2019, 439, 227027.

[45] J.‐I. Lee , K. T. Lee , J. Cho , J. Kim , N.‐S. Choi , S. Park , Angew. Chem., Int. Ed. 2012, 51, 2767. 10.1002/anie.201108915 22307737

[46] H. Tian , X. Tan , F. Xin , C. Wang , W. Han , Nano Energy 2015, 11, 490.

[47] W. Cao , M. Chen , Y. Liu , K. Han , X. Chen , H. Ye , S. Sang , Electrochim. Acta 2019, 320, 134615.

[48] L. Liu , Y. Zhang , N. Xue , Y. Wang , R. Wang , L. Wang , J. Liu , T. Wang , Energy Technol. 2024, 12, 2400664.

[49] W. An , B. Gao , S. Mei , B. Xiang , J. Fu , L. Wang , Q. Zhang , P. K. Chu , K. Huo , Nat. Commun. 2019, 10, 1447. 30926799 10.1038/s41467-019-09510-5 PMC6441089

[50] D. Wang , Y. Ma , W. Xu , S. Zhang , B. Wang , L. Zhi , X. Li , Adv. Mater. 2023, 35, 2212157. 10.1002/adma.202212157 36841944

[51] S. Wei , T. Hartman , S. Mourdikoudis , X. Liu , G. Wang , E. Kovalska , B. Wu , J. Azadmanjiri , R. Yu , L. Chacko , L. Dekanovsky , F. M. Oliveira , M. Li , J. Luxa , S. Jamali Ashtiani , J. Su , Z. Sofer , Adv. Sci. 2024, 11, 2308955. 10.1002/advs.202308955 PMC11199986 38647404

[52] Y. Zhang , W. Tang , H. Gao , M. Li , H. Wan , X. Kong , X. Liu , G. Chen , Z. Chen , ACS Nano 2024, 18, 15671. 38837180 10.1021/acsnano.4c01814

[53] X. Lei , Y. Wang , J. Wang , Y. Su , P. Ji , X. Liu , S. Guo , X. Wang , Q. Hu , L. Gu , Y. Zhang , R. Yang , G. Zhou , D. Su , Small Methods 2024, 8, 2300754. 10.1002/smtd.202300754 37821416

[54] Y. Li , C. Wei , Y. Sheng , F. Jiao , K. Wu , Ind. Eng. Chem. Res. 2020, 59, 12313.

[55] F. Wang , J. Mao , Y. Zhao , Adv. Mater. 2023, 35, 2307908. 10.1002/adma.202307908 37722668

[56] T. Liu , T. Dong , M. Wang , X. Du , Y. Sun , G. Xu , H. Zhang , S. Dong , G. Cui , Nat. Sustain. 2024, 10.1038/s41893-024-01393-9.

[57] K. Kimura , T. Matsumoto , H. Nishihara , T. Kasukabe , T. Kyotani , H. Kobayashi , J. Electrochem. Soc. 2017, 164, A995. 10.1038/srep42734 PMC5316999 28218271

[58] M. Wetjen , S. Solchenbach , D. Pritzl , J. Hou , V. Tileli , H. A. Gasteiger , J. Electrochem. Soc. 2018, 165, A1503.

[59] K. Yao , J. P. Zheng , Z. Liang , ACS Appl. Mater. Interfaces 2018, 10, 7155. 29417815 10.1021/acsami.7b19246

[60] W. He , H. Ji , M. Platonova , R. Chometon , R. Dugas , J.‐M. Tarascon , ACS Appl. Mater. Interfaces 2025, 17, 12125. 39957152 10.1021/acsami.4c20366

[61] X. L. Lin , Y. Lyu , J. Gao , F. Shi , B. Wu , C. F. He , J. Energy Storage 2025, 114, 10.1016/j.est.2025.115896.

[62] A. L. Bhat , J.‐K. Chang , Y.‐S. Su , Electrochim. Acta 2024, 481, 143948.

[63] X. Xiao , W. Zhou , Y. Kim , I. Ryu , M. Gu , C. Wang , G. Liu , Z. Liu , H. Gao , Adv. Funct. Mater. 2015, 25, 1426.

[64] L. Zhang , C. Wang , Y. Dou , N. Cheng , D. Cui , Y. Du , P. Liu , M. Al‐Mamun , S. Zhang , H. Zhao , Angew. Chem., Int. Ed. 2019, 58, 8824. 10.1002/anie.201903709 31050110

[65] N. Liu , H. Wu , M. T. McDowell , Y. Yao , C. Wang , Y. Cui , Nano Lett. 2012, 12, 3315. 22551164 10.1021/nl3014814

[66] J. Wang , L. Liao , Y. Li , J. Zhao , F. Shi , K. Yan , A. Pei , G. Chen , G. Li , Z. Lu , Y. Cui , Nano Lett. 2018, 18, 7060. 30339401 10.1021/acs.nanolett.8b03065

[67] J. Yang , Y.‐X. Wang , S.‐L. Chou , R. Zhang , Y. Xu , J. Fan , W.‐x. Zhang , H. Kun Liu , D. Zhao , S. Xue Dou , Nano Energy 2015, 18, 133.

[68] Y. Li , K. Yan , H.‐W. Lee , Z. Lu , N. Liu , Y. Cui , Nat. Energy 2016, 1, 15029.

[69] X. Zhang , R. Guo , X. Li , L. Zhi , Small 2018, 14, 1800752. 10.1002/smll.201800752 29745010

[70] Z. Li , Z. Zhao , S. Pan , Y. Wang , S. Chi , X. Yi , J. Han , D. Kong , J. Xiao , W. Wei , S. Wu , Q.‐H. Yang , Adv. Energy Mater. 2023, 13, 2300874.

[71] X. Li , Y. Chen , Y. Lu , K. Fu , X. He , K. Sun , J. Ding , Y. Ni , P. Tan , Adv. Funct. Mater. 2024, 10.1002/adfm.202413560.

[72] J. Tao , L. Liu , J. Han , J. Peng , Y. Chen , Y. Yang , H.‐r. Yao , J. Li , Z. Huang , Y. Lin , Energy Storage Mater. 2023, 60, 102809.

[73] M. J. Kim , I. Lee , J. W. Lee , D. Yoon , J. H. Kim , S. Lee , K. Kim , P. J. Kim , J. Choi , Y. C. Kang , D. S. Jung , Small 2024, 20, 2405005. 10.1002/smll.202405005 39308282

[74] H. Wang , Y. Chao , J. Li , Q. Qi , J. Lu , P. Yan , Y. Nie , L. Wang , J. Chen , X. Cui , J. Am. Chem. Soc. 2024, 146, 17041. 38865208 10.1021/jacs.4c01677

[75] D. G. Lee , J. Y. Cho , J. H. Kim , G. Ryoo , J. Yoon , A. Jo , M. H. Lee , J. H. Park , J.‐K. Yoo , D. Y. Lee , J.‐H. Choi , J. T. Han , Adv. Funct. Mater. 2024, 34, 2311353.

[76] Y. Katsuyama , Y. Li , S. Uemura , Z. Yang , M. Anderson , C. Wang , C.‐W. Lin , Y. Li , R. B. Kaner , ACS Appl. Mater. Interfaces 2024, 16, 12612. 38427784 10.1021/acsami.3c18846

[77] M. Hamza , S. Zhang , W. Xu , D. Wang , Y. Ma , X. Li , Nanoscale 2023, 15, 14338. 37581287 10.1039/d3nr02840b

[78] Z. Y. Zhang , Z. W. Li , Q. Luo , B. Z. Yang , Y. Liu , Y. Y. Hu , X. B. Liu , Y. H. Yin , Y. S. Li , Z. P. Wu , Carbon 2022, 188, 238.

[79] Q. Liu , W. Tang , C. Yang , W. Cai , F. Chen , Q. Fu , Chem. Commun. 2023, 59, 7435. 10.1039/d3cc01909h 37254565

[80] Q. Liu , X. Wei , C. Yang , C. Xu , W. Cai , F. Chen , Small 2024, 20, 2403938. 10.1002/smll.202403938 39073236

[81] T. Yang , H. Ying , S. Zhang , J. Wang , Z. Zhang , W.‐Q. Han , Electrochemical Performance Enhancement of Micro‐Sized Porous Si by Integrating with Nano‐Sn and Carbonaceous Materials Materials 2021, [Online]. 10.3390/ma14040920 PMC7919461 33672033

[82] X. Han , L. Gu , Z. Sun , M. Chen , Y. Zhang , L. Luo , M. Xu , S. Chen , H. Liu , J. Wan , Y.‐B. He , J. Chen , Q. Zhang , Energy Environ. Sci. 2023, 16, 5395.

[83] C. Liu , J. Wu , Z. Li , J. Zheng , X. Li , W. Sun , A. Xu , S. Wu , J. Power Sources 2024, 608, 234617.

[84] C. Liu , Q. Xia , C. Liao , S. Wu , Mater. Today Commun. 2019, 18, 66.

[85] Q. Wang , M. Zhu , G. Chen , N. Dudko , Y. Li , H. Liu , L. Shi , G. Wu , D. Zhang , Adv. Mater. 2022, 34, 2109658. 10.1002/adma.202109658 35172027

[86] Z. Zhao , J. Han , F. Chen , J. Xiao , Y. Zhao , Y. Zhang , D. Kong , Z. Weng , S. Wu , Q.‐H. Yang , Adv. Energy Mater. 2022, 12, 2103565.

[87] Z. Liu , Q. Yu , Y. Zhao , R. He , M. Xu , S. Feng , S. Li , L. Zhou , L. Mai , Chem. Soc. Rev. 2019, 48, 285. 30457132 10.1039/c8cs00441b

[88] H. Morimoto , M. Tatsumisago , T. Minami , Electrochem. Solid‐State Lett. 2001, 4, A16.

[89] J. Yang , Y. Takeda , N. Imanishi , C. Capiglia , J. Y. Xie , O. Yamamoto , Solid State Ionics 2002, 152‐153, 125.

[90] M. Zhang , N. Liang , D. Hao , Z. Chen , F. Zhang , J. Yin , Y. Yang , L.‐s. Yang , Nano Res. Energy 2023, 2, 9120.

[91] X. Zhu , B. Liu , J. Shao , Q. Zhang , Y. Wan , C. Zhong , J. Lu , Adv. Funct. Mater. 2023, 33, 2213363.

[92] G. Qian , Y. Li , H. Chen , L. Xie , T. Liu , N. Yang , Y. Song , C. Lin , J. Cheng , N. Nakashima , M. Zhang , Z. Li , W. Zhao , X. Yang , H. Lin , X. Lu , L. Yang , H. Li , K. Amine , L. Chen , F. Pan , Nat. Commun. 2023, 14, 6048. 37770484 10.1038/s41467-023-41867-6 PMC10539371

[93] J. Wang , X. Wang , B. Liu , H. Lu , G. Chu , J. Liu , Y.‐G. Guo , X. Yu , F. Luo , Y. Ren , L. Chen , H. Li , Nano Energy 2020, 78, 105101.

[94] G. Xie , X. Tan , Z. Shi , Y. Peng , Y. Ma , Y. Zhong , F. Wang , J. He , Z. Zhu , X.‐B. Cheng , G. Wang , T. Wang , Y. Wu , Adv. Funct. Mater. 2024, 10.1002/adfm.202414714.

[95] G. Li , L.‐B. Huang , M.‐Y. Yan , J.‐Y. Li , K.‐C. Jiang , Y.‐X. Yin , S. Xin , Q. Xu , Y.‐G. Guo , Nano Energy 2020, 74, 104890.

[96] L.‐B. Huang , L. Zhao , Z.‐F. Ma , X. Zhang , X.‐S. Zhang , Z.‐Y. Lu , G. Li , X.‐X. Luo , R. Wen , S. Xin , Q. Meng , Y.‐G. Guo , Angew. Chem., Int. Ed. 2024, 63, 202413600. 10.1002/anie.202413600 39136072

[97] Y. Zhai , Z. Zhong , N. Kuang , Q. Li , T. Xu , J. He , H. Li , X. Yin , Y. Jia , Q. He , S. Wu , Q.‐H. Yang , J. Am. Chem. Soc. 2024, 146, 15209. 38775661 10.1021/jacs.4c02115

[98] J. Zhong , T. Wang , L. Wang , L. Peng , S. Fu , M. Zhang , J. Cao , X. Xu , J. Liang , H. Fei , X. Duan , B. Lu , Y. Wang , J. Zhu , X. Duan , Nano‐Micro Lett. 2022, 14, 50. 10.1007/s40820-022-00790-z PMC8789978 35076763

[99] Z. Liu , R. Hu , R. Yu , M. Zheng , Y. Zhang , X. Chen , L. Shen , Y. Xia , Nano Lett. 2024, 24, 4908. 10.1021/acs.nanolett.4c00469 38598773

[100] N. Fu , Z. Liu , B. Shen , W. Shao , T. Wang , H. Zhao , J. Wang , Q. Chen , J. Luo , Y. Liu , Z. Yang , Adv. Funct. Mater. 2024, 10.1002/adfm.202410839.

[101] Y.‐F. Tian , G. Li , D.‐X. Xu , Z.‐Y. Lu , M.‐Y. Yan , J. Wan , J.‐Y. Li , Q. Xu , S. Xin , R. Wen , Y.‐G. Guo , Adv. Mater. 2022, 34, 2200672. 10.1002/adma.202200672 35147252

[102] D.‐X. Xu , Y.‐M. Zhao , H.‐X. Chen , Z.‐Y. Lu , Y.‐F. Tian , S. Xin , G. Li , Y.‐G. Guo , Angew. Chem., Int. Ed. 2024, 63, 202401973. 10.1002/anie.202401973 38520059

[103] B. Liu , J. Liu , C. Zhong , W. Hu , Carbon Energy. 2024, 6, 421.

[104] X. Guo , H. Xu , W. Li , Y. Liu , Y. Shi , Q. Li , H. Pang , Adv. Sci. 2023, 10, 2206084. 10.1002/advs.202206084 PMC9896072 36470654

[105] R. Yu , Y. Pan , Y. Liu , L. Zhou , D. Zhao , J. Wu , L. Mai , ACS Nano 2023, 17, 2568. 36646069 10.1021/acsnano.2c10381

[106] Z. Wang , L. Kong , Z. Guo , X. Zhang , X. Wang , X. Zhang , Chem. Eng. J. 2022, 428, 131060.

[107] Y. Zhang , W. Yang , X. Liu , G. Ma , G. Hu , Z. Liu , R. Yu , D. Zhuang , J. Xu , D. Zhao , L. Mai , L. Zhou , Adv. Funct. Mater. 2024, 34, 2315680.

[108] M. Sun , T. Fang , H. Liu , Y. Li , W. Peng , F. Zhang , X. Fan , Chem. Eng. J. 2024, 484, 149359.

[109] G. Wu , Y. Gao , Z. Weng , Z. Zheng , W. Fan , A. Pan , N. Zhang , X. Liu , R. Ma , G. Chen , Carbon Neutralization 2024, 3, 857.

[110] J. Li , K. Yang , Y. Zheng , S. Gao , J. Chai , X. Lei , Z. Zhan , Y. Xu , M. Chen , Z. Liu , Q. Guo , ACS Appl. Mater. Interfaces 2023, 15, 30302. 37337474 10.1021/acsami.3c05103 PMC10317022

[111] Z. Liu , Y. Yang , Q. Zhu , M. Li , B. Xu , Inorg. Chem. Front. 2024, 11, 1511.

[112] Z. Song , S. Chen , Y. Zhao , S. Xue , G. Qian , J. Fang , T. Zhang , C. Long , L. Yang , F. Pan , Small 2021, 17, 2102256. 10.1002/smll.202102256 34528381

[113] L. Yang , T. Meng , W. Zheng , J. Zhong , H. Cheng , Y. Tong , D. Shu , Energy Storage Mater. 2024, 72, 103766.

[114] Y. Zhang , Y. Zhang , X. Wang , H. Gong , Y. Cao , K. Ma , S. Zhang , S. Wang , W. Yang , L. Wang , J. Sun , Adv. Energy Mater. 2024, 10.1002/aenm.202403751.

[115] S. Chen , Z. Deng , J. Li , W. Zhao , B. Nan , Y. Zuo , J. Fang , Y. Huang , Z.‐W. Yin , F. Pan , L. Yang , Angew. Chem., Int. Ed. 2024, 10.1002/anie.202413927. 39304910

[116] L. Dong , H.‐J. Yan , Q.‐X. Liu , J.‐Y. Liang , J. Yue , M. Niu , X. Chen , E. Wang , S. Xin , X. Zhang , C. Yang , Y.‐G. Guo , Angew. Chem., Int. Ed. 2024, 63, 202411029. 10.1002/anie.202411029 38955769

[117] G. Liu , M. Xia , J. Gao , Y. Cheng , M. Wang , W. Hong , Y. Yang , J. Zheng , ACS Appl. Mater. Interfaces 2023, 15, 3586. 36598884 10.1021/acsami.2c17512

[118] H. Feng , J. Deng , Y. Liu , S. Mei , B. Xiang , X. Guo , J. Sun , H. Zhang , T. Li , B. Gao , K. Huo , Chem. Eng. J. 2025, 515, 163518.

[119] Y. Gao , K. Zhang , X. Du , G. Liu , Y. Du , J. Li , Chem. Eng. J. 2025, 508, 160846.

[120] Y. Hao , X. Yang , Y. Gao , M. H. Saleem , J. Li , C. Wu , G. Liu , Chem. Eng. J. 2025, 515, 163258.

[121] W. Yan , S. Ma , Y. Su , T. Song , Y. Lu , L. Chen , Q. Huang , Y. Guan , F. Wu , N. Li , Energy Storage Mater. 2025, 76, 104140.

[122] J. Sun , L. Lv , Y. Li , Y. Wang , L. Wang , W. Xiong , L. Huang , Q. Qu , H. Zheng , Angew. Chem., Int. Ed. 2025, 10.1002/anie.202507688. 40405791

[123] F. Chen , J. Han , D. Kong , Y. Yuan , J. Xiao , S. Wu , D.‐M. Tang , Y. Deng , W. Lv , J. Lu , F. Kang , Q.‐H. Yang , Natl. Sci. Rev. 2021, 8, nwab012. 34691733 10.1093/nsr/nwab012 PMC8433081

[124] C. Wang , H. Wu , Z. Chen , M. T. McDowell , Y. Cui , Z. A. Bao , Nat. Chem. 2013, 5, 1042. 24256869 10.1038/nchem.1802

[125] W. Chen , Z. Zhang , F. Zhang , Z. Wei , P. Li , C. Wang , M. Gu , Y. Deng , J. Chang , Adv. Mater. 2025, 10.1002/adma.202506911. 40401603

[126] J. He , Y. Deng , J. Han , T. Xu , J. Qi , J. Li , Y. Zhang , Z. Zhao , Q. Li , J. Xiao , J. Zhang , D. Kong , W. Wei , S. Wu , Q.‐H. Yang , Nat. Commun. 2025, 16, 4858. 40414886 10.1038/s41467-025-60191-9 PMC12104450

[127] L. Shen , S. Li , Y. Wang , J. Lu , F. Xi , H. Zhao , Z. Tong , W. Ma , Y. Lei , Carbon Energy 2025, 10.1002/cey2.70004.

[128] M. Ma , H. Li , Y. Hu , M. Hamza , W. Cao , Y. Ma , L. Li , X. Li , ACS Appl. Mater. Interfaces 2025, 17, 19657. 40106669 10.1021/acsami.4c22721

[129] C. Yu , X. Chen , Z. Xiao , C. Lei , C. Zhang , X. Lin , B. Shen , R. Zhang , F. Wei , Nano Lett. 2019, 19, 5124. 31260631 10.1021/acs.nanolett.9b01492

[130] Y. Fu , D. Li , X. Sun , Y. Xue , Y. Shi , Z. Li , C. Luo , Q. Lin , X. Gui , K. Xu , Small 2024, 20, 2403070. 10.1002/smll.202403070 38770743

[131] J. Li , S. Zhang , G. Zeng , Z. Xi , M. D. Khan , J. J. Biendicho , A. Cabot , L. Ci , Q. Sun , ACS Appl. Energy Mater. 2025, 8, 7753.

[132] Y. Jia , Y. Qiao , Y. Xu , P. Ji , M. S. Kurbanov , H. Gou , C. Zhang , G. Wang , Carbon 2025, 242, 120445.

[133] X. Liu , J. Zhou , G. Zhu , J. Li , H. Zhang , J. Mater. Chem. A 2025, 10.1039/D5TA03139G.

[134] K. Guo , D. Guo , J. Li , A. Zhou , W. Ding , X. Li , Y. Luo , Y. Wang , S. Lin , G. Liu , N. Wu , X. Liu , A. Qin , J. Mater. Sci. 2025, 60, 9263.

[135] Y. Liu , H. Mu , L. Feng , W. Xin , J. Niu , X. Zhang , Y. Wang , G. Li , ACS Omega 2025, 10, 17642. 40352547 10.1021/acsomega.4c11551 PMC12059954

[136] F. Dou , Y. Chang , Y. Zhang , Q. Li , T.‐Y. Chen , Y. Yan , Y. Sun , X. Guo , C. Yin , H. Zhou , H.‐Y. Chen , H. Pang , Nano Lett. 2025, 25, 8915. 40411527 10.1021/acs.nanolett.5c00832

[137] W. Wen , Y. Liu , A. G. Tamirat , ACS Omega 2025, 10, 21030. 40488090 10.1021/acsomega.4c07644 PMC12138653

[138] J. Liu , H. Zhang , K. Tao , Y. Zhu , S. Li , Y. Hua , H. Songtian , T. Han , J. Li , Chem. Commun. 2025, 61, 7438. 10.1039/d5cc01537e 40277189

[139] T.‐w. Kwon , J. W. Choi , A. Coskun , Chem. Soc. Rev. 2018, 47, 2145. 29411809 10.1039/c7cs00858a

[140] B. Li , P.‐F. Cao , T. Saito , A. P. Sokolov , Chem. Rev. 2023, 123, 701. 36577085 10.1021/acs.chemrev.2c00575

[141] C. Wang , H. Wu , Z. Chen , M. T. McDowell , Y. Cui , Z. Bao , Nat. Chem. 2013, 5, 1042. 24256869 10.1038/nchem.1802

[142] Z. Chen , C. Wang , J. Lopez , Z. Lu , Y. Cui , Z. Bao , Adv. Energy Mater. 2015, 5, 1401826.

[143] T. Munaoka , X. Yan , J. Lopez , J. W. F. To , J. Park , J. B. H. Tok , Y. Cui , Z. Bao , Adv. Energy Mater. 2018, 8, 1703138.

[144] Z. Xu , J. Yang , T. Zhang , Y. Nuli , J. Wang , S.‐i. Hirano , Joule 2018, 2, 950.

[145] C. C. Nguyen , T. Yoon , D. M. Seo , P. Guduru , B. L. Lucht , ACS Appl. Mater. Interfaces 2016, 8, 12211. 27135935 10.1021/acsami.6b03357

[146] F. Wang , Y. Wang , Z. Liu , C. Zhang , L. Li , C. Ye , J. Liu , J. Tan , Adv. Energy Mater. 2023, 13, 2301456.

[147] G. Liu , S. Xun , N. Vukmirovic , X. Song , P. Olalde‐Velasco , H. Zheng , V. S. Battaglia , L. Wang , W. Yang , Adv. Mater. 2011, 23, 4679. 21953600 10.1002/adma.201102421

[148] D. Liu , Y. Zhao , R. Tan , L.‐L. Tian , Y. Liu , H. Chen , F. Pan , Nano Energy 2017, 36, 206.

[149] T. M. Higgins , S.‐H. Park , P. J. King , C. Zhang , N. McEvoy , N. C. Berner , D. Daly , A. Shmeliov , U. Khan , G. Duesberg , V. Nicolosi , J. N. Coleman , ACS Nano 2016, 10, 3702. 26937766 10.1021/acsnano.6b00218

[150] B. Zhang , Y. Dong , J. Han , Y. Zhen , C. Hu , D. Liu , Adv. Mater. 2023, 35, 2301320. 10.1002/adma.202301320 37029618

[151] J. Zhao , J. Jing , W. Li , W. Chen , T. Chen , H. Zhong , Y. Wang , J. Fu , Energy Storage Mater. 2023, 63, 102991.

[152] L. Ding , Y. Zhao , A. Omar , W. Feng , M. Hantusch , D. Mikhailova , Adv. Funct. Mater. 2024, 34, 2305934.

[153] P. Mu , S. Zhang , H. Zhang , J. Li , Z. Liu , S. Dong , G. Cui , Adv. Mater. 2023, 35, 2303312. 10.1002/adma.202303312 37470468

[154] L. Sun , Y. Liu , L. Wang , Z. Jin , Adv. Funct. Mater. 2024, 34, 2403032.

[155] L. Chen , K. Wang , X. Xie , J. Xie , J. Power Sources 2007, 174, 538.

[156] Y.‐F. Tian , S.‐J. Tan , C. Yang , Y.‐M. Zhao , D.‐X. Xu , Z.‐Y. Lu , G. Li , J.‐Y. Li , X.‐S. Zhang , C.‐H. Zhang , J. Tang , Y. Zhao , F. Wang , R. Wen , Q. Xu , Y.‐G. Guo , Nat. Commun. 2023, 14, 7247. 37945604 10.1038/s41467-023-43093-6 PMC10636032

[157] Z.‐h. Chang , X. Li , F.‐l. Yun , Z.‐c. Shao , Z.‐h. Wu , J.‐t. Wang , S.‐g. Lu , ChemElectroChem 2020, 7, 1135.

[158] H. Jia , L. Zou , P. Gao , X. Cao , W. Zhao , Y. He , M. H. Engelhard , S. D. Burton , H. Wang , X. Ren , Q. Li , R. Yi , X. Zhang , C. Wang , Z. Xu , X. Li , J.‐G. Zhang , W. Xu , Adv. Energy Mater. 2019, 9, 1900784.

[159] Y. Liu , Y. Huang , X. Xu , Y. Liu , J. Yang , J. Lai , J. Shi , S. Wang , W. Fan , Y.‐P. Cai , Y.‐Q. Lan , Q. Zheng , Adv. Funct. Mater. 2023, 33, 2303667.

[160] B. Philippe , R. Dedryvère , M. Gorgoi , H. Rensmo , D. Gonbeau , K. Edström , J. Am. Chem. Soc. 2013, 135, 9829. 23763546 10.1021/ja403082s

[161] G. Zeng , Y. An , S. Xiong , J. Feng , ACS Appl. Mater. Interfaces 2019, 11, 23229. 31252474 10.1021/acsami.9b05570

[162] J. Chen , X. Fan , Q. Li , H. Yang , M. R. Khoshi , Y. Xu , S. Hwang , L. Chen , X. Ji , C. Yang , H. He , C. Wang , E. Garfunkel , D. Su , O. Borodin , C. Wang , Nat. Energy 2020, 5, 386.

[163] A.‐M. Li , Z. Wang , T. Lee , N. Zhang , T. Li , W. Zhang , C. Jayawardana , M. Yeddala , B. L. Lucht , C. Wang , Nat. Energy 2024, 10.1038/s41560-024-01619-2.

[164] A.‐M. Li , Z. Wang , T. P. Pollard , W. Zhang , S. Tan , T. Li , C. Jayawardana , S.‐C. Liou , J. Rao , B. L. Lucht , E. Hu , X.‐Q. Yang , O. Borodin , C. Wang , Nat. Commun. 2024, 15, 1206. 38332019 10.1038/s41467-024-45374-0 PMC10853533

[165] K. Doi , Y. Yamada , M. Okoshi , J. Ono , C.‐P. Chou , H. Nakai , A. Yamada , Angew. Chem., Int. Ed. 2019, 58, 8024. 10.1002/anie.201901573 PMC6593729 30951223

[166] Q. Li , J. Ruan , S. Weng , X. Zhang , J. Hu , H. Li , D. Sun , X. Wang , F. Fang , F. Wang , Angew. Chem., Int. Ed. 2023, 62, 202310297. 10.1002/anie.202310297 37697625

[167] Y. Wang , T. Li , X. Yang , Q. Yin , S. Wang , H. Zhang , X. Li , Adv. Energy Mater. 2024, 14, 2303189.

[168] H. J. Rugh , J. Lee , C. Sun , E. E. Abdo , J. N. Bem , N. P. Balsara , G. W. Coates , Angew. Chem., Int. Ed. 2024, 10.1002/anie.202415069. 39414565

[169] Y. Nikodimos , W.‐N. Su , B. J. Hwang , Adv. Energy Mater. 2023, 13, 2202854.

[170] M. Ezzedine , M.‐R. Zamfir , F. Jardali , L. Leveau , E. Caristan , O. Ersen , C.‐S. Cojocaru , I. Florea , ACS Appl. Mater. Interfaces 2021, 13, 24734. 34019366 10.1021/acsami.1c03302

[171] D. H. S. Tan , Y.‐T. Chen , H. Yang , W. Bao , B. Sreenarayanan , J.‐M. Doux , W. Li , B. Lu , S.‐Y. Ham , B. Sayahpour , J. Scharf , E. A. Wu , G. Deysher , H. E. Han , H. J. Hah , H. Jeong , J. B. Lee , Z. Chen , Y. S. Meng , Science 2021, 373, 1494. 34554780 10.1126/science.abg7217

[172] H. Pan , L. Wang , Y. Shi , C. Sheng , S. Yang , P. He , H. Zhou , Nat. Commun. 2024, 15, 2263. 38480726 10.1038/s41467-024-46472-9 PMC10937906

[173] D.‐Y. Han , I. K. Han , J. Y. Kwon , S. Nam , S. Kim , Y. Song , Y. Kim , Y. S. Kim , S. Park , J. Ryu , Adv. Sci. 2025, 12, 2417143. 10.1002/advs.202417143 PMC12165079 40237362

[174] A.‐M. Li , Z. Wang , T. Lee , N. Zhang , T. Li , W. Zhang , C. Jayawardana , M. Yeddala , B. L. Lucht , C. Wang , Nat. Energy 2024, 9, 1551.

[175] Z. He , C. Zhang , Y. Zhu , F. Wei , Energy Environ. Sci. 2024, 17, 3358.

[176] Z. He , Z. Xiao , H. Yue , Y. Jiang , M. Zhao , Y. Zhu , C. Yu , Z. Zhu , F. Lu , H. Jiang , C. Zhang , F. Wei , Adv. Funct. Mater. 2023, 33, 2300094.

[177] B. Peng , Y. Xu , X. Wang , X. Shi , F. M. Mulder , Sci. China: Phys., Mech. Astron. 2017, 60, 064611.

[178] H. Zhong , Y. Su , Y. Wu , J. Gu , R. Ma , Y. Luo , H. Lin , M. Tao , J. Chen , Z. Liang , K. Wang , X. Zheng , Z. Chen , J. Peng , Z. Lv , Z. Gong , J. Huang , Y. Yang , Adv. Energy Mater. 2023, 13, 2300767.

[179] K. G. Araño , B. L. Armstrong , G. Yang , C. Kumara , T. Z. Ward , H. M. Meyer, III , A. M. Rogers , E. Toups , G. M. Veith , Energy Fuels 2024, 38, 6446.

[180] B. Zhang , D. Liu , H. Xie , D. Wang , C. Hu , L. Dai , J. Power Sources 2022, 539, 231591.

[181] G. Lee , Y. Choi , H. Ji , J. Y. Kim , J. P. Kim , J. Kang , O. Kwon , D. W. Kim , J. H. Park , Carbon 2023, 202, 12.

[182] J. Yeon Kim , G. Lee , M. Ryu , J. Hyup Lee , Y. Ji , J. Hyeok Park , D. Woo Kim , Chem. Eng. J. 2024, 497, 154635.

[183] Q. Shi , Y. Cheng , J. Wang , J. Zhou , H. Q. Ta , X. Lian , K. Kurtyka , B. Trzebicka , T. Gemming , M. H. Rümmeli , Small 2023, 19, 2205284. 10.1002/smll.202205284 36433825

[184] L. Ma , Y. Fang , N. Yang , N. Li , L. Chen , D. Cao , Y. Lu , Q. Huang , T. Song , Y. Su , F. Wu , Adv. Mater. 2024, 36, 2404360. 10.1002/adma.202404360 38657134

[185] Q. Shi , J. Zhou , S. Ullah , X. Yang , K. Tokarska , B. Trzebicka , H. Q. Ta , M. H. Rümmeli , Energy Storage Mater. 2021, 34, 735.

[186] R. Baddour‐Hadjean , J.‐P. Pereira‐Ramos , Chem. Rev. 2010, 110, 1278. 19921829 10.1021/cr800344k

[187] P. P. R. M. L. Harks , F. M. Mulder , P. H. L. Notten , J. Power Sources 2015, 288, 92.

[188] J. Yang , N. Solomatin , A. Kraytsberg , Y. Ein‐Eli , ChemistrySelect 2016, 1, 572.

[189] Q. Fang , S. Xu , X. Sha , D. Liu , X. Zhang , W. Li , S. Weng , X. Li , L. Chen , H. Li , B. Wang , Z. Wang , X. Wang , Energy Environ. Sci. 2024, 17, 6368.

[190] Y. Gao , L. Fan , R. Zhou , X. Du , Z. Jiao , B. Zhang , Nano‐Micro Lett. 2023, 15, 222. 10.1007/s40820-023-01190-7 PMC10562352 37812292

[191] H. Jia , X. Li , J. Song , X. Zhang , L. Luo , Y. He , B. Li , Y. Cai , S. Hu , X. Xiao , C. Wang , K. M. Rosso , R. Yi , R. Patel , J.‐G. Zhang , Nat. Commun. 2020, 11, 1474. 32193387 10.1038/s41467-020-15217-9 PMC7081208

[192] X. Gao , W. Lu , J. Xu , J. Power Sources 2020, 449, 227501.

[193] P. Xue , R. Qiu , C. Peng , Z. Peng , K. Ding , R. Long , L. Ma , Q. Zheng , Adv. Sci. 2024, 10.1002/advs.202410065. PMC11672295 39556707

[194] Z. Huang , L. Sugiarto , Y.‐C. Lu , EcoMat. 2023, 5, 12345.

[195] F. Fu , X. Wang , L. Zhang , Y. Yang , J. Chen , B. Xu , C. Ouyang , S. Xu , F.‐Z. Dai , E. W. , Adv. Funct. Mater. 2023, 33, 2303936.

[196] Y. Luo , Y. Chen , N. Koratkar , W. Liu , Adv. Sci. 2024, 11, 2403530. 10.1002/advs.202403530 PMC11425885 38975809

[197] N. Kim , Y. Kim , J. Sung , J. Cho , Nat. Energy 2023, 8, 921.

[198] Z. Chen , Y. Luo , D. Yang , Y. Hu , H. Hou , N. Koratkar , G. Zhou , W. Liu , Mater. Today 2025, 86, 74.

[199] Y. Deng , C. Li , R. Guo , Z. Xie , L. Huang , J. He , L. Xing , W. Li , Adv. Funct. Mater. 2025, 35, 2415820.

[200] X. Zeng , X. Liu , H. Zhu , J. Zhu , J. Lan , Y. Yu , Y.‐S. Lee , X. Yang , Adv. Energy Mater. 2024, 14, 2402671.

